# Influence of isopropylmalate synthase *OsIPMS1* on seed vigour associated with amino acid and energy metabolism in rice

**DOI:** 10.1111/pbi.12979

**Published:** 2018-07-16

**Authors:** Yongqi He, Jinping Cheng, Ying He, Bin Yang, Yanhao Cheng, Can Yang, Hongsheng Zhang, Zhoufei Wang

**Affiliations:** ^1^ The Laboratory of Seed Science and Technology State Key Laboratory of Crop Genetics and Germplasm Enhancement Jiangsu Collaborative Innovation Center for Modern Crop Production Nanjing Agricultural University Nanjing China; ^2^ The Laboratory of Seed Science and Technology Guangdong Key Laboratory of Plant Molecular Breeding State Key Laboratory for Conservation and Utilization of Subtropical Agro‐Bioresources South China Agricultural University Guangzhou China

**Keywords:** isopropylmalate synthase, seed vigour, seed priming, *Oryza sativa*

## Abstract

Seed vigour is an imperative trait for the direct seeding of rice. Isopropylmalate synthase (IPMS) catalyses the committed step of leucine (Leu) biosynthesis, but its effect on seed vigour remains unclear. In this study, rice *OsIPMS1* and *OsIPMS2* was cloned, and the roles of *OsIPMS1* in seed vigour were mainly investigated. OsIPMS1 and OsIPMS2 catalyse Leu biosynthesis, and Leu feedback inhibits their IPMS activities. Disruption of *OsIPMS1* resulted in low seed vigour under various conditions, which might be tightly associated with the reduction of amino acids in germinating seeds. Eleven amino acids that associated with stress tolerance, GA biosynthesis and tricarboxylic acid (TCA) cycle were significantly reduced in *osipms1* mutants compared with those in wide type (WT) during seed germination. Transcriptome analysis indicated that a total of 1209 differentially expressed genes (DEGs) were altered in *osipms1a* mutant compared with WT at the early germination stage, wherein most of the genes were involved in glycolysis/gluconeogenesis, protein processing, pyruvate, carbon, fructose and mannose metabolism. Further analysis confirmed that the regulation of *OsIPMS1* in seed vigour involved in starch hydrolysis, glycolytic activity and energy levels in germinating seeds. The effects of seed priming were tightly associated with the mRNA levels of *OsIPMS1* in priming seeds. The *OsIPMS1* might be used as a biomarker to determine the best stop time‐point of seed priming in rice. This study provides novel insights into the function of *OsIPMS1* on seed vigour and should have practical applications in seed priming of rice.

## Introduction

Rice (*Oryza sativa* L.) is one of the most important food crops in the world. Recently, the direct‐seeding method of rice is becoming increasingly popular in China because of its low cost and operational simplicity (Wang *et al*., 2011). High seed vigour, including rapid, uniform germination and vigorous seedling growth, is essential for the direct seeding of rice (Mahender *et al*., 2015). Seeds with speed and uniform germination may significantly improve field emergence, lead to better suppression of weed growth and produce high yield under various conditions (Foolad *et al*., 2007; Wang *et al*., 2010). Therefore, the mining of key genes controlling seed vigour and illuminating their molecular mechanisms are important objectives of rice breeding.

Seed germination is a quantitative trait controlled by multiple genes and environmental factors during seed development and germination stages. To date, several quantitative trait loci (QTLs) for seed germination have been reported in rice (Cheng *et al*., 2013; Fujino *et al*., 2004, 2008; Hsu and Tung, 2015; Li *et al*., 2013; Miura *et al*., 2002; Wang *et al*., 2010, 2011). Of those, several QTLs for germination speed are likely to coincide with QTLs for seed weight, seed size and seed dormancy in rice (Wang *et al*., 2010). The first clone‐related QTL, *qLTG3‐1*, which controls germination speed under various conditions, is tightly associated with vacuolation of tissues covering the embryo (Fujino *et al*., 2008); *qLTG3‐1* plays an important role in the weakening of seed tissues during germination through programmed cell death (Fujino and Matsuda, 2010). The second clone‐related QTL *Sdr4* is associated with seed dormancy, which is positively regulated by *OsVP1* in rice (Sugimoto *et al*., 2010). *OsVP1* is orthologous to *Arabidopsis ABI3*, which is a central component of the abscisic acid (ABA) signalling pathway during seed germination (Graeber *et al*., 2012; Holdsworth *et al*., 2008). The gibberellin (GA) biosynthesis‐related gene *OsGA20ox1* has been reported as a candidate gene for a major QTL controlling seedling vigour (Abe *et al*., 2012). It is widely accepted that the balance between ABA and GA is important for seed germination (Graeber *et al*., 2012; Nambara *et al*., 2010). These results suggest that germination speed is associated with seed weight, size and seed dormancy and is also influenced by endosperm weakening, hormones and storage metabolism (Bethke *et al*., 2007; Catusse *et al*., 2008; Fait *et al*., 2006).

Seed germination and subsequent seedling growth need large amounts of energy and nutrition, which are provided only by seed reserves, because the germinating seeds lack a mineral uptake system and photosynthetic apparatus (Bewley, 1997). When quiescent dry seeds imbibe water, their oxygen uptake increases (Pergo and Ishii‐Iwamoto, 2011) and three respiratory pathways, including glycolysis, pentose phosphate pathway and tricarboxylic acid (TCA) cycle, are activated in the imbibed seeds (Bewley *et al*., 2013). Glycolysis operates under aerobic and anaerobic conditions to produce pyruvate. In the presence of O_2_, further utilization of pyruvate occurs within the mitochondria; that is, oxidative decarboxylation of pyruvate produces acetyl‐CoA, which is completely oxidized to CO_2_ and water via the TCA cycle to yield up to 30 ATP molecules per glucose molecule respired (Bewley *et al*., 2013). In the glycolysis pathway, glyceraldehyde‐3‐phosphate (Gly‐3‐P) dehydrogenase (Gly‐3‐PDH) converts Gly‐3‐P into 1,3‐bisphosphoglycerate, and pyruvate dehydrogenase converts pyruvate into acetyl‐CoA (Xu *et al*., 2016a). Accumulation of glycolytic enzymes in several species, such as Gly‐3‐PDH (Han *et al*., 2014; Kim *et al*., 2009) and pyruvate dehydrogenase (Kim *et al*., 2008) in rice, fructose‐1,6‐bisphosphatase in *Arabidopsis* (Rajjou *et al*., 2008), and Gly‐3‐PDH in sugar beet (Catusse *et al*., 2011) and *Arabidopsis* (Rajjou *et al*., 2008), is positively correlated with seed vigour. Taken together, these results indicate that glycolysis and TCA cycle provide most of the energy for seed germination.

Amino acids are not only used for the synthesis of storage proteins but are also catabolized for the TCA cycle to generate energy (Galili *et al*., 2014). Leucine (Leu) is one of the essential branched‐chain amino acids (BCAA; leucine, valine and isoleucine), serving as an alternative energy source for mammals (Harper *et al*., 1984) and plants (Binder, 2010). For example, Leu promotes energy metabolism (glucose uptake, mitochondrial biogenesis and fatty acid oxidation) for improving protein synthesis while inhibiting protein degradation in mammals (Duan *et al*., 2016). Catabolism of Leu produces energy‐rich intermediates, such as acetyl‐CoA and propionyl‐CoA (Anderson *et al*., 1998). Similarly, BCAA degradation provides energy for seed germination at the early stage in *Arabidopsis* (Ding *et al*., 2012; Gipson *et al*., 2017). α‐Isopropylmalate synthase (IPMS) catalyses the committed step of Leu biosynthesis, that is converting acetyl‐CoA and α‐ketoisovalerate into α‐isopropylmalate (Zhang *et al*., 2014). Overexpression of the IPMS gene (*BatIMS*) from *Brassica* in *Arabidopsis* resulted in plants with an aberrant phenotype, perturbed amino acid metabolism and enhanced levels of glucosinolates (Field *et al*., 2006). A knockout insertion mutant of *Arabidopsis* for *IPMS1* demonstrated an increase in Val levels but no changes in Leu levels; two insertion mutants for *IPMS2* did not show any changes in the soluble amino acid levels (de Kraker *et al*., 2007). Recently, proteomic analysis indicated that IPMS involved in seed dormancy release in rice (Xu *et al*., 2016b).

The activation of amino acid biosynthesis and/or recycling pathways is essential for seed germination (Arc *et al*., 2012). Ketogenic amino acid, for example Leu, isoleucine (Ile), lysine (Lys), phenylalanine (Phe), tryptophan (Trp), tyrosine (Tyr) and threonine (Thr), can be degraded directly into acetyl‐CoA, which is the precursor for GA biosynthesis via hydroxymethylglutaryl‐CoA (Rios‐Iribe *et al*., 2011). It suggests that Lue facilitating not only energy production but also GA biosynthesis in germinating seeds might be important for seed germination. In this study, rice isopropylmalate synthase *OsIPMS1* was cloned, and its effect on seed vigour was mainly investigated. Disruption of *OsIPMS1* resulted in low seed vigour under various conditions, which might be tightly associated with the reduction of amino acid biosynthesis in germinating seeds. The amino acids that associated with stress tolerance, GA biosynthesis and TCA cycle were significantly reduced in *osipms1* mutants compared to wide type (WT) during seed germination. After that, the glycolytic activity and energy levels were significantly decreased in germinating seeds of *osipms1* mutants, resulting low seed vigour in *osipms1* mutants. Our results indicated that the *OsIPMS1* gene might be used to determine the best stop time‐point of seed priming in rice.

## Results

### Characterization of *OsIPMS1* and *OsIPMS2*

Two IPMS genes located on chromosomes 11 and 12 are found in rice. We renamed these two genes as follows: LOC_Os11g04670.1, *OsIPMS1* and LOC_Os12g04440.1, *OsIPMS*2. OsIPMS1 and OsIPMS2 were predicted to contain approximately 635 amino acids, each, which includes an N‐terminal catalytic region and a C‐terminal allosteric regulatory domain (Figure S1a). The phylogenetic tree indicated that the closer genetic relationships of IPMS exist among rice, maize and sorghum; bootstrap value is approximately 70% at branch point (Figure S1b). The sequence showed 99% amino acid identity between OsIPMS1 and OsIPMS2 (Figure S2), suggesting that these two genes may behave similar functions.

**Figure 1:**
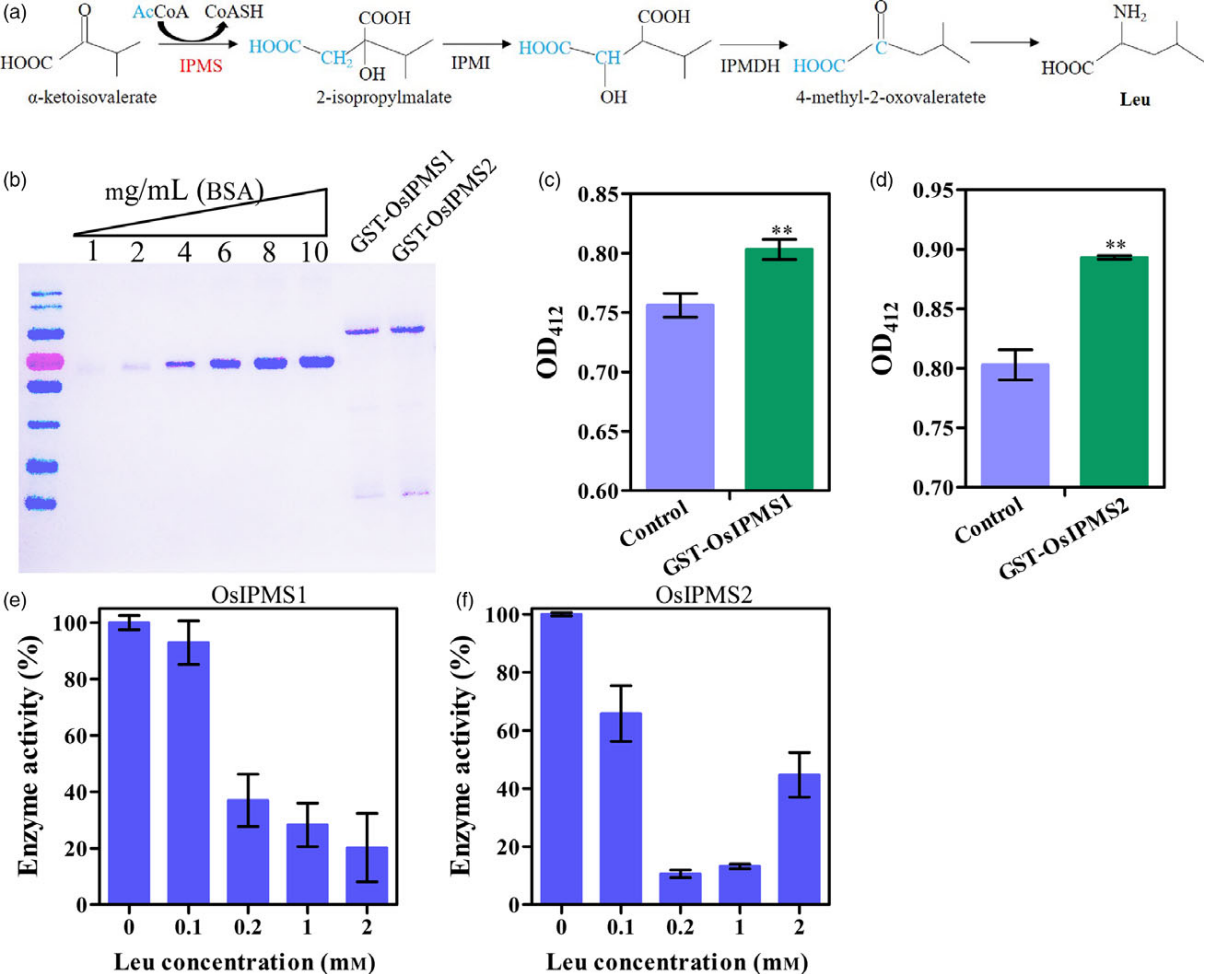
Activities of OsIPMS1 and OsIPMS2 in rice. (a) Pathway for Leu biosynthesis. (b) Purification of the expressed GST‐Tag OsIPMS1 and OsIPMS2 protein. (c, d) OsIPMS1 and OsIPMS2 activities were identified by directly measuring absorbance at 412 nm. (e,f) The activities of OsIPMS1 and OsIPMS2 are subjected to Leu feedback inhibition. ** indicates the significant difference at 1% level.

Isopropylmalate synthase catalyses the committed step of Leu biosynthesis, and it is subject to Leu feedback inhibition (Figure 1a). To test whether OsIPMS1 and OsIPMS2 regulate IPMS activity, the proteins of OsIPMS1 and OsIPMS2 were expressed and extracted (Figure 1b). The output of CoASH was increased with the reaction, which indicated that the OsIPMS1 and OsIPMS2 convert acetyl‐CoA and α‐ketoisovalerate into 2‐isopropylmalate and CoASH (Figure 1c,d). OsIPMS1 and OsIPMS2 activities were significantly declined with the increasing concentrations of Leu (Figure 1e,f), suggesting that the OsIPMS1 and OsIPMS2 enzymes are subject to Leu feedback inhibition.

### Expression pattern of *OsIPMS1* and *OsIPMS2*

**Figure 2:**
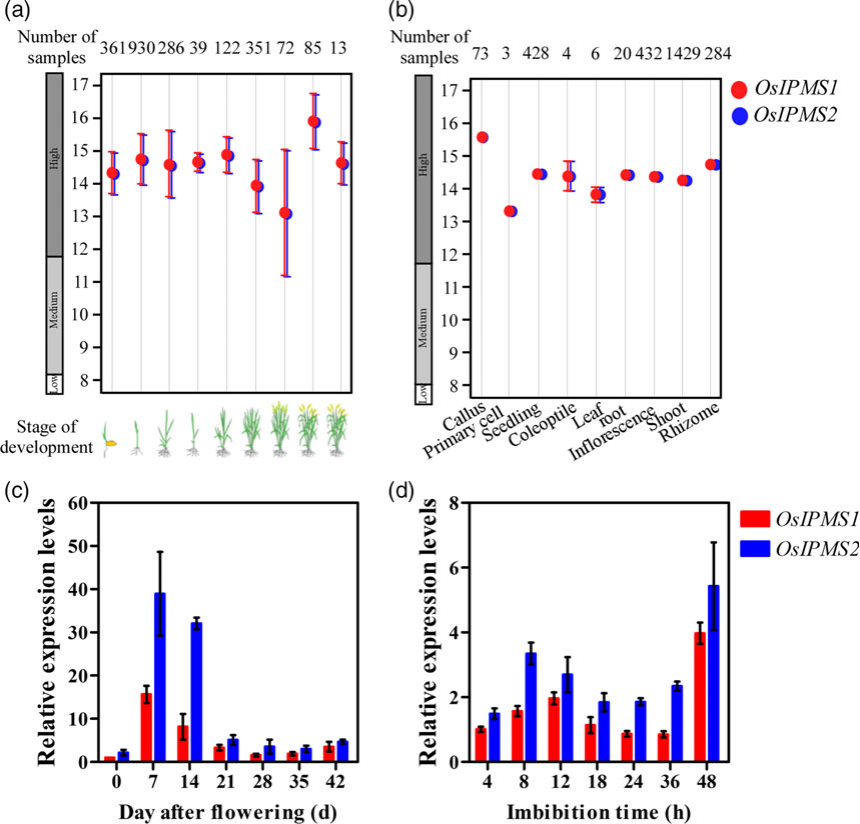
Expression patterns of *OsIPMS1* and *OsIPMS2* in rice. Expression pattern of *OsIPMS1* and *OsIPMS2* in various developmental stages (a) and tissues (b) of rice based on the publicly available microarray data ( http://www.genevestigator.com). Transcription levels of *OsIPMS1* and *OsIPMS2* in filling grains (c) and germinating seeds (d) were conducted using qRT‐PCR approach. The expression of *OsIPMS1* and *OsIPMS2* was normalized to that of *OsActin* gene control. The relative expression levels were represented by fold change relative to the expression level of *OsIPMS1* at 0 DAF (c) or 4‐h imbibition stage (d). Each column represents the means ± standard deviation.

Based on the publicly available microarray database ( http://www.genevestigator.com), the higher transcript abundances of *OsIPMS1* and *OsIPMS2* were observed at all of the developmental stages and in various tissues (Figure 2a,b). To expand our understanding of the physiological function of *OsIPMS1* and *OsIPMS2*, the expression patterns of *OsIPMS1* and *OsIPMS2* during seed development and seed germination were further analysed using qRT‐PCR approach. Transcripts of *OsIPMS1* and *OsIPMS2* were significantly increased in the filling grain from 0 to 7 days after flowering (DAF) and then sharply decreased after the 14th DAF (Figure 2c). Additionally, the transcript levels of *OsIPMS1* and *OsIPMS2* were slightly altered during the 4‐ to 36‐h imbibition stage; however, the transcript was strongly detected at 48‐h imbibition when seeds begin germinating (Figure 2d). Similar spatial‐temporal expression patterns were observed in *OsIPMS1* and *OsIPMS2*, suggesting both genes might behave similar functions in seed germination of rice.

### Disruption of *OsIPMS1* resulted in low seed vigour under various conditions

**Figure 3:**
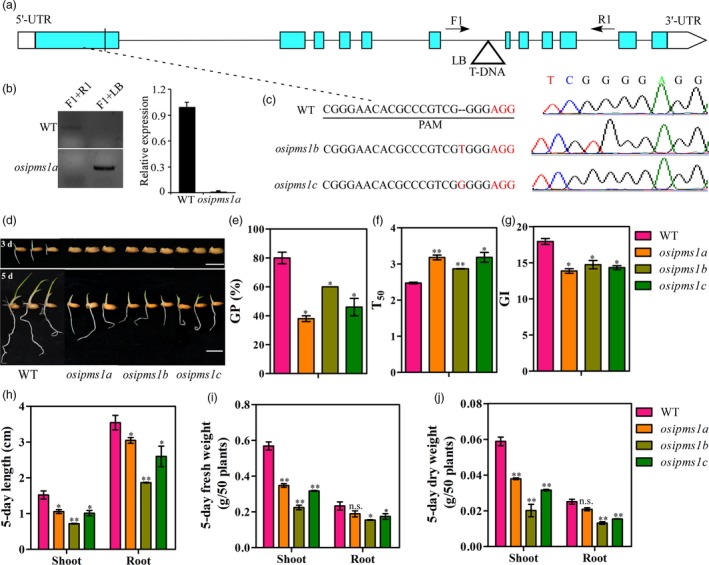
Comparison of seed germination between WT and *osipms1* mutants under normal conditions. (a) Gene structure of *OsIPMS1* with a T‐DNA insertion and locations of the primers used for PCR analysis. Triangle represents the T‐DNA. Green and white boxes represent exons and UTR of *OsIPMS1*, respectively. Solid lines represent introns. F, R and T‐DNA‐F primers used for genotyping PCR. (b) PCR of genomic DNA from WT and *osipms1a*. qRT‐PCR analysis of *OsIPMS1* in WT and *osipms1a* seeds. The expression of *OsIPMS1* was normalized to that of *OsActin* gene control. The relative expression levels were represented by fold change relative to the expression level of WT. (c) One nucleotide was added in the *osipms1b* and *osipms1c* mutants and produced a premature stop. (d) Seed germination of WT and *osipms1* mutants after 3 and 5 days. Bars = 10 mm. (e) germination potential; (f) time to 50% germination percentage; (g) germination index; (h) length of shoots and roots; (i) fresh weight of shoots and roots; (j) dry weight of shoots and roots. Each column represents the means ± standard deviation. * and ** indicate the significant difference compared to WT at 5% and 1% levels, respectively. n.s. represents not significant.

To study the role of *OsIPMS1* on seed vigour, a T‐DNA mutant *osipms1* was subsequently obtained from Rice Mutant Database ( http://rmd.ncpgr.cn/; Miyao *et al*., 2003). The T‐DNA insertion was located in the sixth intron of *OsIPMS1* (Figure 3a). Using specified primer sets (Table S1), a homozygous insertion mutant individual was isolated and named *osipms1a*. Furthermore, RT‐PCR analysis demonstrated that the T‐DNA insertion resulted in a complete suppression of *OsIPMS1* expression in the *osipms1a* mutant (Figure 3b). To further confirm OsIPMS1 function, we employed the CRISPR/Cas9 system to generate mutants, which were named *osipms1b* and *osipms1c*. The *osipms1b* and *osipms1c* mutant plants contained a ‘T’ and ‘G’ insertion in the first exon of *OsIPMS1* (Figure 3c). The levels of *OsIPMS1* expression were much lower in *osipms1b* and *osipms1c* mutants than those in WT plants (Figure S3a). The amino acid sequence of OsIPMS1, predicted based on these nucleotide sequences, contains only 172 amino acids in *osipms1b* and *osipms1c* and is caused by premature termination (Figure S3b). These results indicate that the *osipms1b* and *osipms1c* mutant lines lacked OsIPMS1. The progeny of these homozygous mutants were used in subsequent experiments.

Phenotype evaluation demonstrated that the disruption of *OsIPMS1* resulted in low germination speed and seedling growth under various conditions. The GP and GI of mutant lines (*osipms1a*,* osipms1b* and *osipms1c*) were significantly reduced while T_50_ was significantly increased compared to those of WT plants under normal conditions (Figure 3d–g). The early seedling growth, including the length, fresh and dry weight of shoots and roots, in mutants were also significantly reduced compared with those of WT plants (Figure 3h–j). Similar results were observed in the *osipms1* mutant lines under salt, drought and cold stress conditions (Figure S4). Moreover, seedling emergence and seedling growth were significantly decreased in *osipms1* mutant lines compared to those in WT plants under the direct‐seeding method in soils (Figure S5). Similarly, the significant decreases in germination speed and seedling growth were also observed in *osipms2* mutant lines compared to those in WT plants under normal condition (Figure S6). It further confirmed that *OsIPMS1* and *OsIPMS2* might behave the similar functions in seed germination. Here, the following analyses were mainly focused on the physiological roles of *OsIPMS1* in seed vigour.

### *OsIPMS1* increased amino acid biosynthesis during seed germination

**Figure 4:**
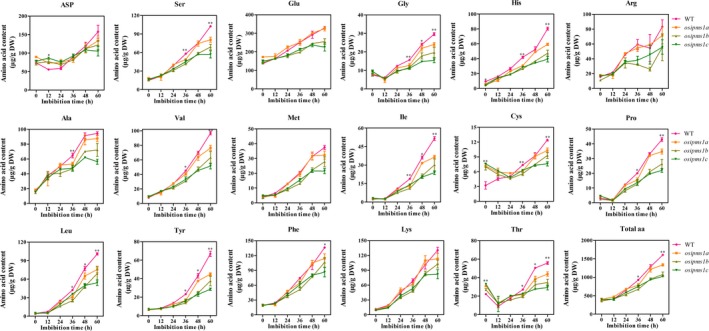
Comparison of amino acid levels between WT and *osipms1* mutants during seed germination. Each point represents the means ± standard deviation. * and ** indicate the significant difference compared to WT at 5% and 1% levels, respectively.

We predicted that the disruption of *OsIPMS1* affects amino acid biosynthesis due to its effect on the IPMS enzyme through Lue biosynthesis. Therefore, the levels of free amino acids in *osipms1* mutants (*osipms1a*,* osipms1b* and *osipms1c*) and WT lines were measured during seed germination (Figure 4). By comparison, the levels of amino acids Thr and cysteine (Cys) were significantly increased in mature unstratified seeds of *osipms1* mutants compared to those in WT plants. However, the decreases in amino acid levels were generally observed in *osipms1* mutants compared to the levels in WT plants during seed germination. In which, the levels of eleven amino acids were significantly decreased in 60‐h imbibed seeds of three *osipms1* mutants simultaneously compared to the levels in WT plants. Approximately 10%–50% lower levels of Leu, Ile, Phe, Tyr, Thr, Cys, serine (Ser), glycine (Gly), valine (Val), histidine (His) and proline (Pro) were observed in mutants than those in WT plants.

**Figure 5:**
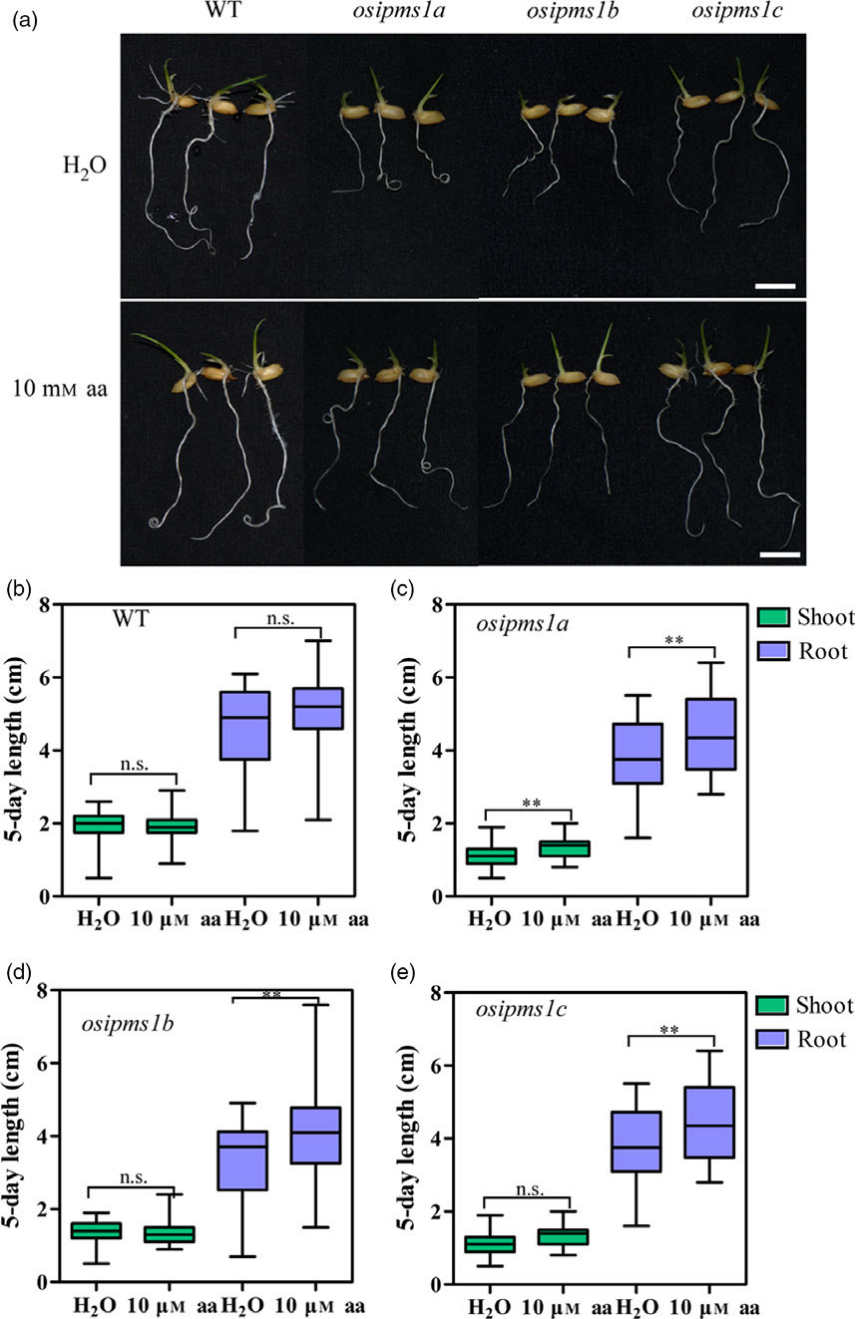
Comparison of seedling growth between WT and *osipms1* mutants under amino acids treatments. (a) Seedling growth in WT and *osipms1* mutants under normal and amino acids treatment conditions for 5 days. Bars = 10 mm. Comparison of shoot and root length between normal and amino acids treatment in WT (b) and *osipms1* mutants (c, d, e). Each column represents the means ± standard deviation. ** indicates the significant difference compared to normal condition at 1% level. n.s. represents not significant.

Overall, the levels of all amino acids were progressively enhanced during seed germination, wherein the amino acids might be used for the synthesis of proteins, hormones and energy for seed germination at the early stages. Higher levels of eleven amino acids in the WT might contribute towards seed germination and vigorous seedling growth in this study. To confirm this hypothesis, the effects of 10 mm amino acids, including Leu, Ile, Phe, Ser, Gly and Val, treatment on seed germination of *osipms1* mutants were determined. We observed that seedling growth of *osipms1* mutants was nearly rescued by exogenous application of amino acids compared to that in WT plants (Figure 5a). Meanwhile, the root lengths of three *osipms1* mutants were significantly increased by amino acids treatment compared to those in untreated seeds (Figure 5b–e). Ketogenic amino acid, for example Leu, Ile, and Phe, can be degraded directly into acetyl‐CoA for GA biosynthesis (Rios‐Iribe *et al*., 2011). Here, we predicted that amino acid treatment contributing to seed vigour of *osipms1* mutants might be through stimulating GA biosynthesis during seed germination. Expectedly, the expressions of GA biosynthesis‐related genes in *osipms1* mutants were significantly increased after amino acid treatment compared with those in the control (Figure S7). These results suggest that the regulation of *OsIPMS1* on seed germination might be through altering amino acids associated with GA biosynthesis in germinating seeds.

### Disruption of *OsIPMS1* altered gene expression during seed germination

To further understand the *OsIPMS1* function, genomewide transcriptional levels were compared between *osipms1a* and WT at the 8‐h imbibition stage. A total of 1209 differentially expressed genes (DEGs) were identified between *osipms1a* and WT (Figure S8; Table S2). Of those, 424 and 785 genes were significantly up‐regulated and down‐regulated, respectively, in *osipms1a* compared to WT. GO and KEGG pathway categories of DEGs were examined. Most overrepresented GO categories were classified as biological processes, including single‐organism process, single‐organism metabolic process and carbohydrate metabolic process (Figure S8). Most overrepresented KEGG pathway categories were related to glycolysis/gluconeogenesis, protein processing and carbon, pyruvate and fructose metabolism, etc (Table S3). Of these, 27 and 31 genes were associated with glycolysis/gluconeogenesis and proteins processing, respectively, and 13 and 29 genes were associated with pyruvate and carbon metabolism.

**Figure 6:**
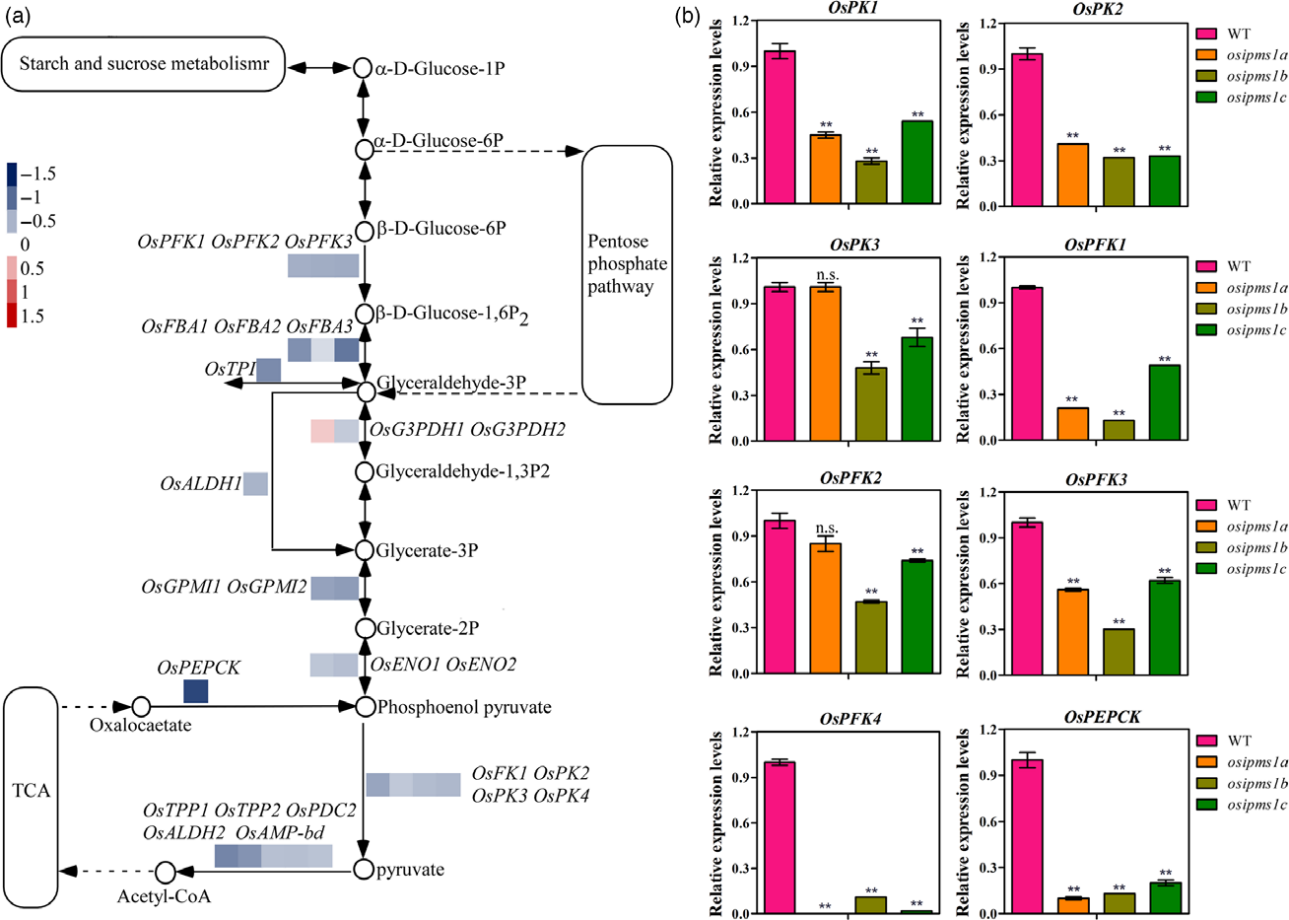
*OsIPMS1* altering expression of genes involved in glycolysis. (a) Differentially expressed genes (DEGs) involved in glycolysis. (b) Comparison of *OsPKs*,* OsPFKs* and *OsPEPCK* expression between WT and *osipms1* at 8‐h imbibition stage using qRT‐PCR approach. The expression of genes was normalized to that of *OsActin* gene control. The relative expression levels were represented by fold change relative to the expression level of WT. Each metabolism column represents the means ± standard deviation. ** indicates the significant difference compared to WT at 1% level. n.s. represents not significant.

GO and KEGG analysis indicated that the altering of glycolysis process is an important factor in *OsIPMS1* regulation of seed germination. To confirm this hypothesis, qRT‐PCR was used to determine the transcript levels of *OsPFK* and *OsPK* genes, which are two key regulated genes in glycolysis (Figure 6a). Consistent alterations in the levels of these genes were observed through RNA sequencing and qRT‐PCR approaches. The transcripts of *OsPKs*,* OsPFKs* and *OsPEPCK* were significantly reduced in *osipms1* compared to those in WT at 8‐h seed germination stage (Figure 6b). These data suggest that germination regulated by *OsIPMS1* is tightly associated with glycolytic metabolism.

### *OsIPMS1* enhanced starch mobilization during seed germination

Imbibition is the critical first step to induce starch hydrolysis for glycolysis during seed germination. Therefore, dynamic imbibition was compared between mutants and WT during seed germination (Figure S9a). A significant decrease in the imbibition rate was only observed at the seed germinating stage (60 h) when the radicle protruded in mutants compared to that of WT. This outcome suggests that the altering of germination by *OsIPMS1* is not through the influence of imbibition rate at the early germination stage. The initial imbibition is primarily affected by protein and starch levels in mature seeds. We observed that there were no significant differences in total protein and starch levels in mature seeds of mutants compared to those in WT, whereas the total soluble sugar levels were significantly decreased in mutants (Figure S9b–d). These results indicate that the disruption of *OsIPMS1* reduced seed vigour might be partly due to decreasing total soluble sugar levels in mature seeds.

**Figure 7:**
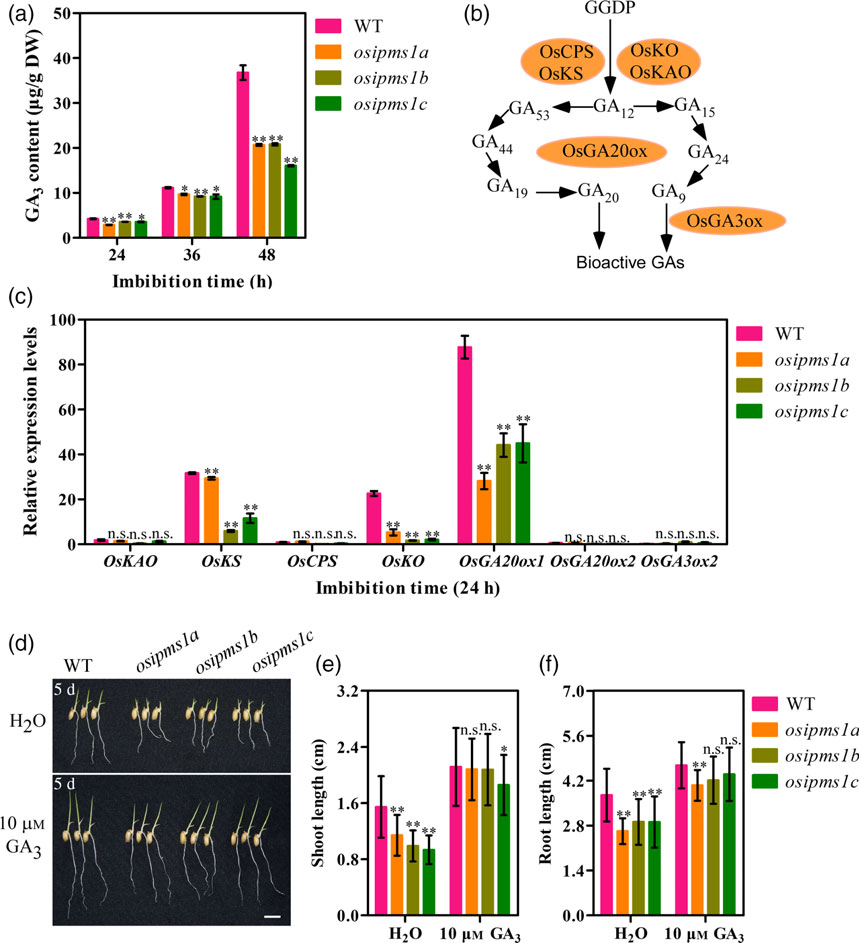
*OsIPMS1* altering seedling growth involved in GA biosynthesis. (a) GA_3_ content in WT and *osipms1* mutants during seed germination. (b) General overview of GA biosynthesis pathway in rice according to previous reports. (c) Relative expression levels of GA biosynthesis‐related genes in germinating seeds. The expression of genes was normalized to that of *OsActin* gene control. The relative expression levels were represented by fold change relative to the expression level of *OsKAO* in WT. (d) Seedling growth in WT and *osipms1* mutants under normal and GA_3_ treatment conditions for 5 days. Bars = 10 mm. Comparison of shoot length (e) and root length (f) between WT and *osipms1* mutants under normal and GA_3_ treatment conditions. Each column represents the means ± standard deviation. * and ** indicate the significant difference compared to WT at 5% and 1% levels, respectively. n.s. represents not significant.

**Figure 8:**
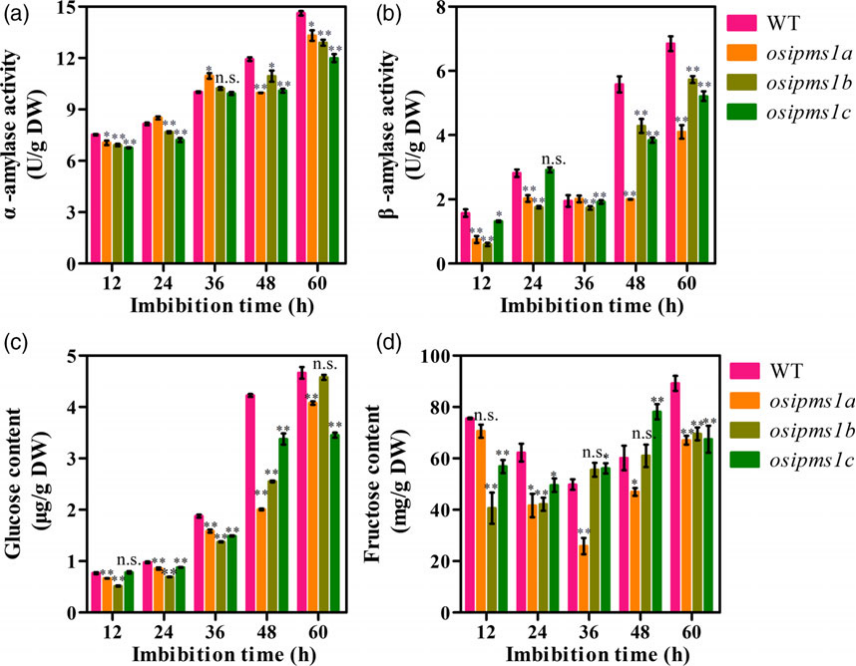
Comparison of starch mobilization between WT and *osipms1* mutants during seed germination. (a) α‐amylase activity; (b) β‐amylase activity; (c) glucose content; (d) fructose content. Each column represents the means ± standard deviation. * and ** indicate the significant difference compared to WT at 5% and 1% levels, respectively. n.s. represents not significant.

Starch hydrolysis by amylase is an important contributing factor for seed germination and vigorous seedling growth. The α‐amylase biosynthesis is induced by GA during seed germination. As described above, the amino acids associated with GA biosynthesis, including Leu, Ile, Phe, Thr and Tyr, were significantly reduced in three *osipms1* mutants compared to those in WT; the treatment of amino acid can increase the expression of GA biosynthesis‐related genes in germinating seeds of *osipms1* mutants. Thus, we predicted that GA biosynthesis might be regulated by *OsIPMS1* in germinating seeds. Expectedly, the levels of GA_3_ were significantly reduced in *osipms1* mutants compared to those in WT during seed germination (Figure 7a). The expressions of GA biosynthesis‐related genes, including *OsKS*,* OsKO* and *OsGA20ox1*, were significant decreased in *osipms1* mutants compared with those in WT (Figure 7b,c). It was also confirmed by the analysis of GA_3_ treatments on seed germination of *osipms1* mutants. We observed that seedling growth of *osipms1* mutants was nearly rescued by exogenous application of GA_3_ compared to those in WT (Figure 7d,e,f). Furthermore, we observed that the activities of α‐amylase and β‐amylase (Figure 8a,b), and the levels of glucose and fructose were generally and significantly lower in mutants than those in WT during seed germination (Figure 8c,d). These results indicate that *OsIPMS1* regulates seed vigour might be through altering starch hydrolysis in germinating seeds.

### *OsIPMS1* improved glycolytic activity and ATP level during seed germination

**Figure 9:**
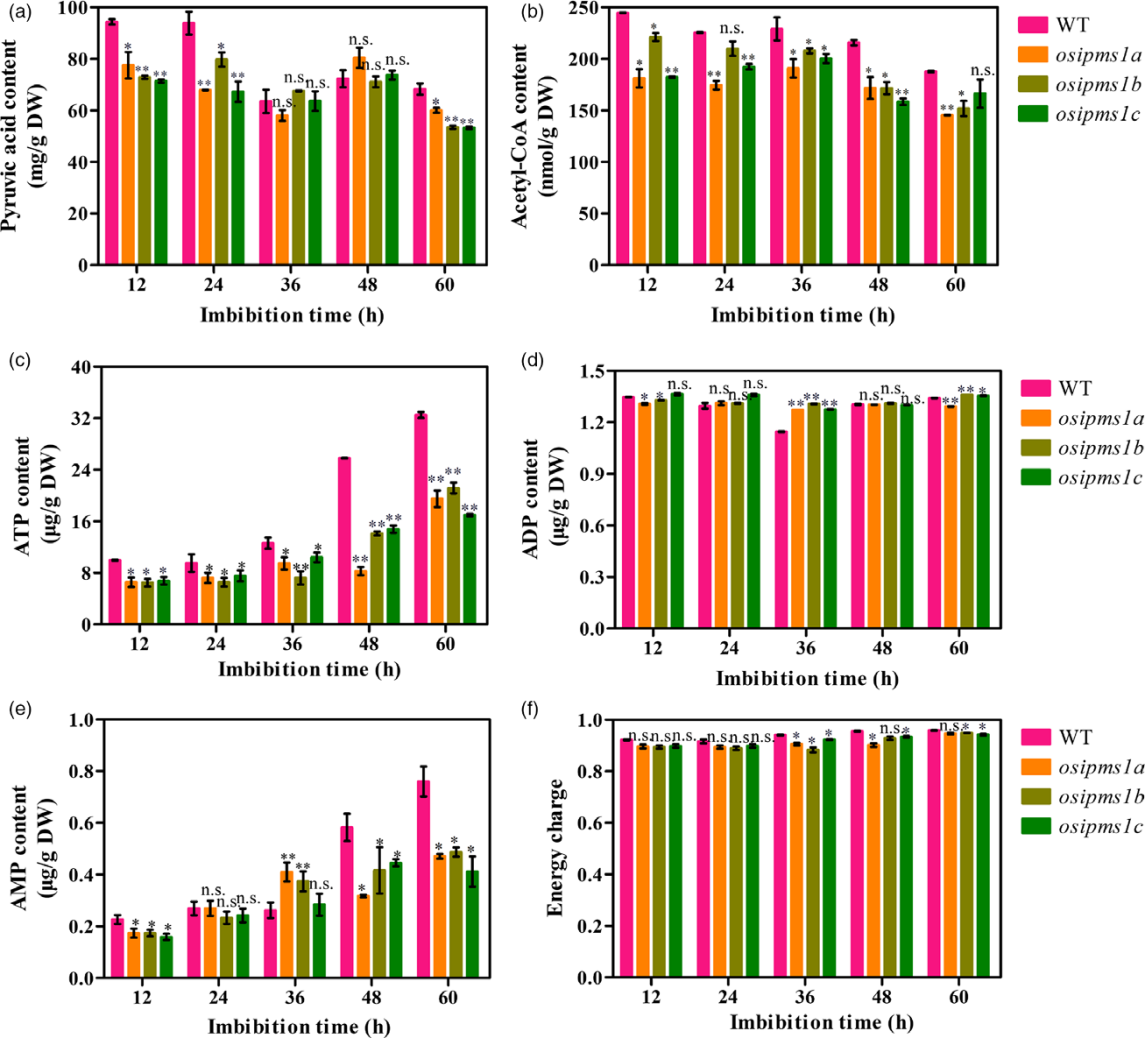
Comparison of glycolytic activity and energy levels between WT and *osipms1* mutants during seed germination. (a) pyruvate acid content; (b) acetyl‐CoA content; (c) ATP content; (d) ADP content; (e) AMP content; (f) energy charge. Each column represents the means ± standard deviation. * and ** indicate the significant difference compared to WT at 5% and 1% levels, respectively. n.s. represents not significant.

To further examine the regulation of glycolytic activity by *OsIPMS1*, the intermediate metabolites of glycolysis were assayed during seed germination. Generally, the levels of pyruvate and acetyl‐CoA were significantly decreased in *osipms1* mutants compared to those in WT (Figure 9a,b). This outcome suggests that glycolytic activity was reduced in *osipms1* mutants compared to that in WT. The intermediate metabolites of glycolysis are associated with energy production during seed germination. We observed that the levels of ATP, AMP and energy charge were significantly reduced in *osipms1* mutants compared to those in WT during seed germination (Figure 9c–f). Notably, the ATP and AMP levels in *osipms1* mutants were decreased by approximately 50% compared to those in WT at the late germination stage (48 and 60 h). These results further demonstrate that *OsIPMS1* is involved in the regulation of glycolysis and energy production during seed germination.

### Application of *OsIPMS1* for seed priming

Seed priming is a technique used to improve seed vigour, which allows imbibition to a certain extent but prevents radicle emergence. Thus, an important requirement for successful seed priming is to stop the priming treatment and dehydrate the seed at the right moment (Paparella *et al*., 2015). To determine whether *OsIPMS1* expression influencing priming effect, the different duration of priming treatments were conducted and compared between *osipms1* mutants and WT. The significant higher GI and lower T_50_ were observed in 12‐h primed seeds compared to those in unprimed seeds (0 h) in WT plants (Figure S10). Less adverse effects of priming were observed in 24‐ and 36‐h primed seeds in WT; however, the adverse effects of priming were observed in 12‐, 24‐ and 36‐h primed seeds in *osipms1* mutants. Generally, the significant lower GP, GI and SP but higher T_50_ were observed in 12‐, 24‐ and 36‐h primed seeds in *osipms1* mutants compared to those in unprimed seeds. It suggests that the disruption of *OsIPMS1* will reduce the priming effects in rice.

**Figure 10:**
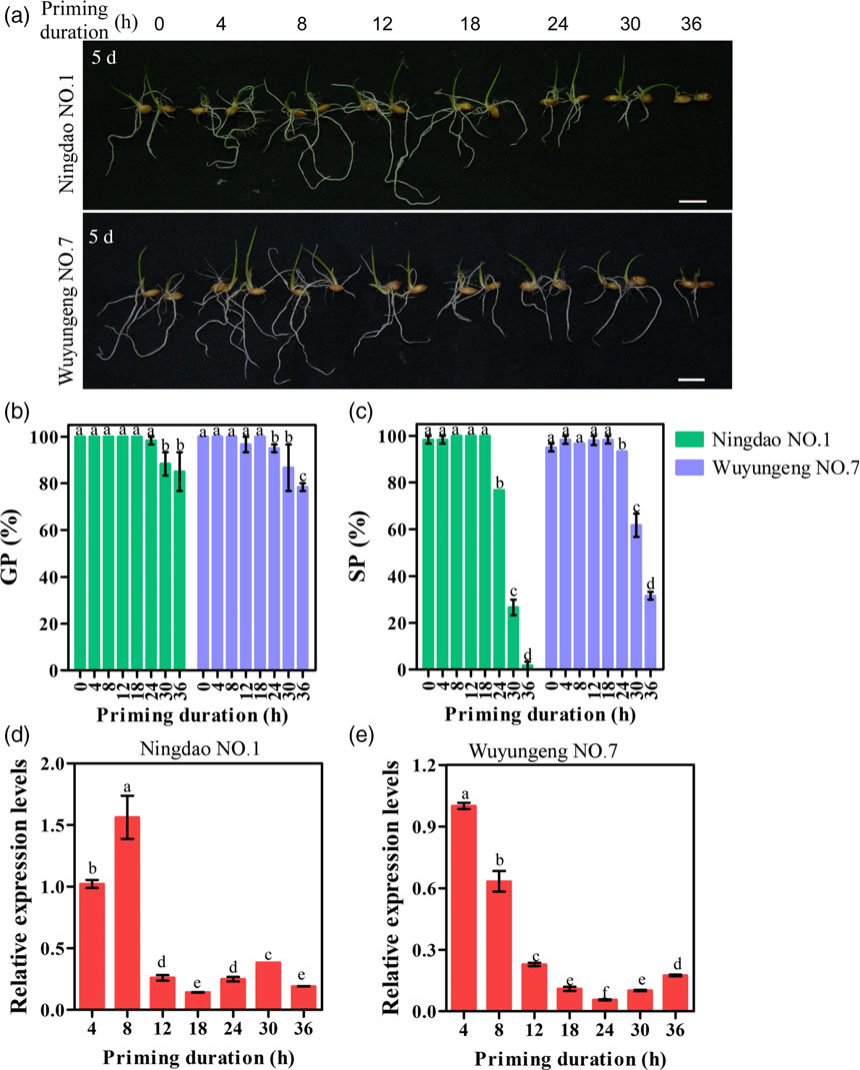
Relationship between priming effects and *OsIPMS1* expression in primed seeds of rice Ningdao NO.1 and Wuyungeng NO.7. (a) Comparison of seedling growth among various durations of priming treatments. Bars = 10 mm. (b) germination potential; (c) seedling percentage. (d,e) Transcription levels of *OsIPMS1* during the priming process. The expression of *OsIPMS1* was normalized to that of *OsActin* gene control. The relative expression levels were represented by fold change relative to the expression level of *OsIPMS1* at 4‐h priming stage. Each column represents the means ± standard deviation. Different lowercase letters represent significant difference at 5% level.

To further determine the correlation between priming effects and the mRNA levels of *OsIPMS1* during seed priming, various durations of priming treatments were conducted using rice cv. Ningdao NO.1 and Wuyungeng NO.7. Generally, the better seedling growth was observed in 4‐, 8‐ and 12‐h primed seeds compared to that in unprimed seeds, but the adverse effects of priming were observed during 18‐ to 36‐h priming treatments (Figure 10a). The significant decreases in GP, SP and seedling growth were observed in 24‐, 30‐ and 36‐h primed seeds compared to those in unprimed seeds (Figure 10a–c). By comparison, the best priming effects, including GP, SP and seedling growth, were observed in 8‐h primed seeds in both rice varieties. qRT‐PCR analysis showed that the relative higher mRNA levels of *OsIPMS1* were observed in 4‐ and 8‐h priming seeds, but the mRNA levels were significantly reduced in seeds after 12‐h priming treatments (Figure 10d,e). These results suggest that the best stop time‐point of seed priming might be at the time before the mRNA levels of *OsIPMS1* significantly reduced in priming seeds.

## Discussion

Seed vigour is an important agronomic trait that consists of seed longevity, germination and seedling growth, etc. However, the trait of seed vigour has not been selected in conventional breeding due to its complex nature and quantitative inheritance in rice. Identification and utilization of vigour‐related genes are important for the improvement of seed vigour in rice. Previous studies demonstrated that IPMS catalyses the committed step of Leu biosynthesis (Zhang *et al*., 2014). There have been few reports regarding the phenotype of *IPMS* disruption in plants (Field *et al*., 2006). The present study demonstrated that the disruption of *OsIPMS1* and *OsIPMS2* reduced seed vigour in rice. To the best of our knowledge, this is the first report highlighting the involvement of *IPMS* regulation in seed vigour of plants. The roles of *OsIPMS1* influencing seed germination were analysed in this study, and the application of *OsIPMS1* as a biomarker for seed priming was also discussed in rice.

Isopropylmalate synthase contains two core domains – an N‐terminal catalytic region and a C‐terminal allosteric regulatory domain – which mediate Leu feedback inhibition in plants and bacteria (de Kraker and Gershenzon, 2011). Similar results were observed in this study. OsIPMS1 and OsIPMS2 catalyse Leu biosynthesis, and generally their IPMS activities were gradually inhibited with the increase in Leu concentration in rice. However, the OsIPMS2 activity was activated by 2 mm Leu treatment in this study, which similar with the observation in *Arabidopsis* (de Kraker *et al*., 2007). OsIPMS1 and OsIPMS2 might have similar but not identical biochemical characteristics, and this prediction need to be further confirmed. We observed that eleven amino acids, including Leu and Val, were significantly decreased in the three *osipms1* mutants during seed germination. This outcome was in contrast to the results involving *Arabidopsis*, wherein the *IPMS1* mutant showed an increase in the Val levels but no changes in the Leu levels (de Kraker *et al*., 2007). Moreover, the decreases in soluble sugars in mature seeds were observed in *osipms1* mutants compared to that in WT in this study. Soluble sugars, for example glucose and sucrose, serve as the primary energy source for seed germination (Ding *et al*., 2012). We speculated that the regulation of *OsIPMS1* in seed vigour might be associated with soluble sugars in mature seeds. The roles of *OsIPMS1* controlling amino acids biosynthesis and seed quality during seed development are deserved to further investigation.

It is well known that seed germination is linked to numerous amino acid metabolic pathways. Reduction in Cys synthesis induces an irreparable loss of seed vigour (Rajjou *et al*., 2008). Pro accumulation is effective in improving seed germination and seedling growth under stress conditions (Biju *et al*., 2017). Similarly, we observed that Cys and Pro synthesis were significantly decreased in *osipms1* mutants compared with those in WT. Cys and Pro are important features of seed vigour owing to their general implications in metabolism and antioxidative potential in plants (Rajjou *et al*., 2008). Additionally, we observed that the branched‐chain amino acids (BCAAs), including Leu, Ile and Val, were significantly reduced in *osipms1* mutants compared to those in WT. BCAAs biosynthesis plays an important role in gametophyte and root development, and BCAA homeostasis contributes to stress tolerance in plants (Zhang *et al*., 2015). We therefore predicted that *OsIPMS1* contributing to seed vigour might be due to the enhancement of amino acids associated with stress tolerance in germinating seeds. The relationships between amino acids, for example Cys, Pro, Ile, Val and Leu, and stress tolerance during seed germination need to be further analysed.

Initial seed imbibition is characterized by physical water uptake (Bewley *et al*., 2013). Usually, proteinaceous seeds have higher imbibition capacities than those of starch and oily seeds at the early germination stage. In this study, similar imbibition rates were observed at the early germination stage in *osipms1* mutants and WT due to the similar protein and starch levels in their mature seeds. These results indicated that *OsIPMS1* regulates seed vigour but not by altering the imbibition rate. GA biosynthesis upon seed imbibition is necessary for seed germination and seedling growth (Vieira *et al*., 2002). In this study, we observed that the amino acids related to GA biosynthesis were significantly reduced in *osipms1* mutants compared to those in WT. Further amino acid and GA treatments confirmed that the regulation of *OsIPMS1* on seed vigour was significantly associated with GA biosynthesis in germinating seeds. GA stimulates the expression of α‐amylase genes for hydrolysis of carbohydrate reserves, and then nourishes seed germination and seedling growth (Vieira *et al*., 2002). In this study, the decline of soluble sugars was observed in *osipms1* mutants compared to those in WT during seed germination. It suggests that the regulation of *OsIPMS1* in seed vigour might be involved in the accumulation of soluble sugars served as the primary energy source for seed germination (Ding *et al*., 2012).

Glycolysis is critical for germination and seedling growth as it generates energy. By comparison, the glycolytic pathway appears to be much more affected by seed vigour than other pathways (Wang *et al*., 2015). The regulation of *OsIPMS1* on seed vigour might be tightly associated with the glycolysis in this study. This hypothesis was confirmed by the global analysis of transcripts in imbibed seeds, wherein glycolysis was expected to be highly inactive in *osipms1a* mutants compared to that in WT. It was further confirmed by qRT‐PCR analysis of genes encoding the rate limiting enzymes of glycolysis, including phosphofructokinase and pyruvate kinase. Phosphofructokinase and pyruvate kinase are important control points in the glycolytic pathway, as they catalyse two of the irreversible steps. Pyruvate kinase catalyses the final step of glycolysis, in which pyruvate and ATP are formed. Expressions of these genes were significantly decreased in *osipms1a* mutants compared to those in WT. Meanwhile, we observed that the expression of other glycolytic genes, such as fructose‐bisphosphate aldolase isozyme, aldehyde dehydrogenase, thiamine pyrophosphate enzyme, Gly‐3‐PDH and pyruvate decarboxylase, was also regulated in *osipms1a* mutants. These results are in alignment with reports wherein the glycolytic enzymes Gly‐3‐PDH (Han *et al*., 2014; Kim *et al*., 2009) and pyruvate dehydrogenase (Kim *et al*., 2008) were demonstrated to be associated with seed vigour in rice.

Amino acids serve as energy donors in the TCA cycle under night‐time, developmental and stress conditions in plants (Angelovici *et al*., 2011; Araújo *et al*., 2010, 2011; Kirma *et al*., 2012). The aspartic acid (Asp) family pathway synthesizes the amino acids Lys, Thr, Ile and methionine (Met), which are further catabolized by the TCA cycle to generate energy under energy shortage conditions (Galili, 2011). The relationship between amino acids, for example Lys and Asp, and the TCA cycle has been observed during the early seed germination (Araújo *et al*., 2011). In this study, we observed that the ketogenic amino acids, including Leu, Ile, Phe, Cys, Gly, Thr and Tyr, were significantly reduced in *osipms1* mutants compared to those in WT. These ketogenic amino acids are important for pyruvate and acetyl‐CoA synthesis, which are the starting substrates of the TCA cycle. Metabolism of His and Pro is important for the synthesis of the TCA cycle metabolite, α‐ketoglutarate. Similarly, metabolism of Iso, Val and Thr also has important effects on the synthesis of the TCA cycle metabolite, succinate, whereas Phe and Tyr metabolism are important for fumarate synthesis. However, these amino acids, including His, Pro, Iso, Val, Thr, Phe and Tyr, were significantly decreased in *osipms1* mutants compared to those in WT in this study. These amino acids altered by *OsIPMS1* might behave important effects on the TCA cycle during seed germination in rice.

As description above, we hypothesized that the *OsIPMS1* may exert its effects on seed vigour by altering the energy level during seed germination. This hypothesis was firstly confirmed by the analysis of pyruvate and acetyl‐CoA levels during seed germination. Pyruvate is the product of glycolysis, which is converted into acetyl‐CoA, which in turn is the primary input for the TCA cycle (Schwender *et al*., 2003). We observed that the levels of pyruvate and acetyl‐CoA were significantly decreased in *osipms1* mutants compared to those in WT during seed germination. Pyruvate dehydrogenase catalysing the conversion of pyruvate to acetyl‐CoA is a step linking the glycolytic pathway to the TCA cycle (Wang *et al*., 2015). In this study, decreased expression of pyruvate dehydrogenase was also observed in *osipms1* mutants, suggesting that the carbon flux through the TCA cycle declined. Finally, the levels of ATP and AMP dropped approximately 30% in *osipms1* mutants compared to those in WT at the late germination stage. These data implied that disruption of *OsIPMS1* will reduce energy levels in germinating seeds, potentially causing low seed vigour in *osipms1* mutants.

The positive effects of priming on seed vigour are mainly due to triggering the metabolic processes when primed seeds rehydration, including the activation of DNA repair and antioxidant mechanisms, de novo synthesis of nucleic acids and proteins and ATP production (Paparella *et al*., 2015). Our data demonstrated that the expression of *OsIPMS1* contributes to seed germination associated with ATP production. This raises the question as to the association between priming effects and *OsIPMS1* expression during priming. It is difficult to choose and monitor the correct time‐point of stop priming and dehydrate the seeds (Paparella *et al*., 2015). Meanwhile, the success of seed priming is strongly associated with plant species, genotype, seed lot and vigour (Parera and Cantliffe, 1994). Thus, two varieties Ningdao NO.1 and Wuyungeng NO.7 that popularly cultivated in Jiangsu Province of China were used to confirm the association between priming effects and *OsIPMS1* expression during priming in this study. We observed that the best stop time‐point of seed priming is likely at the time before the mRNA levels of *OsIPMS1* significantly reduced during priming, suggesting a suitable extent of germination‐related metabolism existed in primed seeds at that moment. Prolonged priming duration caused a significantly low mRNA level of *OsIPMS1* stored in primed seeds that will cause low seed vigour when primed seeds rehydration. Previously, prolonged priming treatment reduced seed vigour has been ascribed to the loss of seed desiccation tolerance (Sliwinska and Jendrzejczak, 2002) and an unpredicted enhancement of oxidative DNA injury (Balestrazzi *et al*., 2010). In this study, we predicted that prolonged priming treatment reduced the priming effects is possibly due to low ATP production in primed seeds when rehydration. The confirmation of this hypothesis is now in progress. The *OsIPMS1* might be used as a biomarker to determine the best time‐point of stop priming in rice. However, the different expression of *OsIPMS1* was also observed among varieties. The applications of *OsIPMS1* in seed priming need to be further investigated using more varieties at more time‐points of priming in rice.

**Figure 11:**
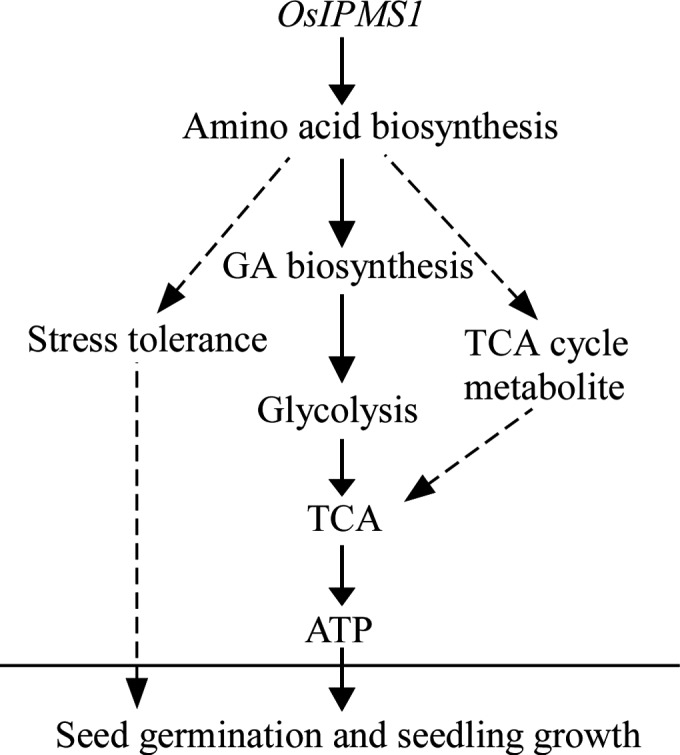
Hypothetical model of the role of *OsIPMS1* on seed vigour in rice. The expression of *OsIPMS1* in germinating seeds induces the accumulation of the amino acids associated with stress tolerance, GA biosynthesis and TCA cycle. Increased GA biosynthesis enhances starch hydrolysis for the accumulation of soluble sugars, thus increasing the glycolytic activity. The ATP levels will be promoted through TCA cycle with the increases of glycolysis and TCA cycle metabolites, which contribute to rapid germination and vigorous seedling growth. Solid arrows indicate the major effects, while dashed arrows indicate the predicted minor effects.

In summary, the regulation of *OsIPMS1* in seed vigour might be involved in the changes of amino acids that associated with stress tolerance, GA synthesis and TCA cycle in germinating seeds (Figure 11). The improvement of amino acids associated with stress tolerance might be contributing to seed germination under various conditions. Meanwhile, *OsIPMS1* can promote the synthesis of amino acids related to GA biosynthesis in germinating seeds, which results in the enhancement of soluble sugars for glycolysis during seed germination. After that, the ATP levels will be promoted through TCA cycle with the increases in glycolysis and TCA cycle metabolites, which contribute to rapid germination and vigorous seedling growth. This study provides important insights into the function of *OsIPMS1* on seed vigour in rice.

## Materials and methods

### Plant materials and growth conditions

The *osipms1* (*osipms1a*,* osipms1b* and *osipms1c*) and *osipms2* mutants used in this study were in the *Japonica* background (*Oryza sativa* L. cv. Nipponbare). The T‐DNA mutant *osipms1a* (accession number: 05NPBMT72) was obtained from Rice Mutant Database ( http://rmd.ncpgr.cn/; Miyao *et al*., 2003); *osipms1b*,* osipms1c* and *osipms2* mutants were generated using the CRISPR/Cas9 system. All plants were grown in an experimental field at the Nanjing Agricultural University. Field management was performed in accordance with the local standard methods (Cheng *et al*., 2014). All seeds were harvested at their maturity stage and dried at 42 ^°^C for 7 days (∼13% moisture content) to break seed dormancy (Wang *et al*., 2011).

### T‐DNA mutant identification

The T‐DNA insertion site and homozygous line of *osipms1a* was identified by PCR analysis of genomic DNA using gene‐specific primers (Table S1). PCR was conducted according to Xu *et al*. (2017). PCR products were directly sequenced and compared using NCBI BLAST of the rice genome database ( http://www.ncbi.nlm.nih.gov/Blast/). Quantitative real‐time PCR (qRT‐PCR) analysis, as described below, was conducted to detect the mRNA levels of *OsIPMS1* in 48‐h imbibed seeds of *osipms1a* compared to that in the wild type (WT, Nipponbare).

### Mutant generation and identification

The CRISPR/Cas9 system was used to generate mutants. The CRISPR/Cas9 plasmid was designed according to the protocol described previously (Cong and Zhang, 2015). The first coding exon of *OsIPMS1* and *OsIPMS2* was selected for the guide RNA design. Double‐stranded DNA, generated by annealing the oligo pairs, was cloned into the p1300 + pX330‐U6‐Chimeric_BB‐CBh‐hSpCas9 vector. Genomic DNA was extracted from mutant seedlings for PCR using specific primers (Table S1). Mutations in the PCR products were detected through direct sequencing methods. Next, the PCR products were identified by comparing the 20‐bp gRNA target sequences to the rice reference genome (sequence is in Table S1; Liu *et al*., 2015). qRT‐PCR analysis, as described below, was conducted to detect the mRNA levels in 48‐h imbibed seeds of mutants compared to those in WT. Three independent homozygous mutants, *osipms1b*,* osipms1c* and *osipms2*, were identified.

### Expression and purification of GST‐Tag Protein

The sequence data of *OsIPMS1* and *OsIPMS2* were obtained from the Institute for Genomic Research (TIGR) database. The coding sequence of *OsIPMS1* and *OsIPMS2* was amplified from the reverse‐transcribed RNA isolated in rice Nipponbare seedlings using the primers IPMS1‐1S, IPMS1‐1A, IPMS2‐1S and IPMS2‐1A (Table S1). The cDNA fragments were ligated into the pGEM‐T easy vector (Promega, Madison, WI). The desired *OsIPMS1* and *OsIPMS2* fragments were amplified by PCR using the primers IPMS1‐2S, IPMS1‐2A, IPMS2‐2S and IPMS2‐2A (Table S1), and subcloned into pGEX‐2T using the restriction sites BamH I and EcoR I. The glutathione‐S‐transferase (GST)‐IPMS fusion proteins were expressed in *E. coli* BL21 (DE3) cells and grown in LB medium at 37 °C until they reached an OD_600_ of 0.5. The GST‐IPMS1 proteins were then induced with 0.5 mM isopropyl‐1‐thio‐β‐D‐galactopyranoside (IPTG) and incubated with constant shaking at 15 °C for 24 h. Protein expression was checked by SDS‐PAGE analysis. Proteins were purified using a GST•BindTM Kit (Novagen, Germany) and quantified with gel densitometry using a bovine serum albumin (BSA, Sigma‐Aldrich) protein standard.

### OsIPMS enzyme assay

The OsIPMS1 and OsIPMS2 enzyme assay was conducted according to the methods of de Kraker *et al*. (2007) with minor modifications. Briefly, 5 μL enzyme preparation was added into 150 μL reaction mixture, including 500 mM acetyl‐CoA, 10 mm 2‐oxoisovalerate, 4 mm MgCl_2_ and 100 mm Tris, pH 8.0, and incubated at 30 °C for 10 min. Reactions were stopped using liquid nitrogen. Next, 200 μL 1 mm fresh DTNB (Sigma‐Aldrich) in 100 mm Tris (pH 8.0), and 200 μL ethanol was added into the reaction mixture. The mixture was left at room temperature (20–25 °C) until no further reaction occurred between the free thiol group of CoA with DNTB to develop a yellow‐coloured 3‐carboxy‐4‐nitrothiophenol anion. The mixture was centrifuged, and the absorbance was detected against water at 412 nm. The amount of OD_412_ was used to indicate enzyme activity. Three biological replications were performed.

### Seed germination

Fifty seeds per replicate were imbibed in Petri dishes (diameter 9 cm) with 10 mL distilled water, 10% PEG or 100 mm NaCl at 30 ± 1 °C for 7 days, as well as with 10 mL distilled water at 15 ± 1 °C for 14 days. Thirty seeds per replicate were sowed in 1 cm deep soils under natural conditions (25–32 °C) for 7 days. Meanwhile, seed germination of *osipms1* mutants was also conducted under GA_3_ and amino acids treatments. Germination ability was observed daily. Seeds were considered as germinated when the radicle protruded (2 mm) through the seed coat. Seedlings were considered to be established when the root length reached seed length and the shoot length reached half of the seed length. The percentage of germinated seeds at 3 days was referred to as germination potential (GP). T_50_ is the time for 50% of the germination, and it was calculated by the GERMINATOR software (Joosen *et al*., 2010). Germination index (GI) was calculated as follows: GI = ∑ (*Gt*/*t*), where *Gt* is the number of the germinated seeds on Day *t* (Wang *et al*., 2010). Three replications were performed.

### Expression analysis

Total RNA was extracted from various plant tissues of Nipponbare, as well as from developing grains (0, 7, 14, 21, 28, 35 and 42 days after flowering; DAF) and germinating seeds (4‐, 8‐, 12‐, 18‐, 24‐, 36‐ and 48‐h imbibition), using the TransZol Plant kit (Transgen, www. transgen.com), according to the protocol by the manufacturer. The first‐strand cDNA was synthesized with random oligonucleotides using the HiScript^®^ II Reverse Transcriptase system (Vazyme Biotech Co., Ltd). qRT‐PCR was carried out in a total volume of 20 μL containing 2 μL of cDNA, 0.4 μL gene‐specific primers (10 μm), 10 μL SYBR Green Mix and 7.2 μL of RNase free ddH_2_O, using the Roche LightCycler480 Real‐time System (Roche, Swiss Confederation). The PCR conditions were as follows: 95 ^°^C for 5 min, followed by 40 cycles of 95 °C for 15 s and 60 °C for 30 s. The rice *OsActin* and *18S rRNA* genes were used as internal controls. Primers used for qRT‐PCR are listed in Table S1. Normalized transcript levels were calculated using the comparative CT method (Livak and Schmittgen, 2001). Three biological replications were performed.

### Differentially expressed genes analysis

Total RNA was extracted from approximately 80~100 mg powder of WT and *osipms1* seeds after 8‐h imbibition using the TransZol Plant kit (Transgen, www.transgen.com) according to the manufacturer's protocol. Construction of cDNA libraries and HiSeq2500 sequencing were performed at Novogene Biotechnology Co., Ltd., Beijing, China. FastQC was performed to estimate the quality of raw reads ( http://www.plob.org/2013/07/16/5987.html). The adapter sequences were trimmed, and the low‐quality reads with *Q* ≤ 20 from the 5′ and 3′ ends of the remaining reads were filtered. Then, the clean reads with 21–49 bp length were mapped onto the Nipponbare reference genome (MSU Rice Genome Annotation Project Release 7) using Tophat version 2.0.12 (Kim *et al*., 2012). Levels of gene expression were quantified in terms of FPKM (fragments per kilo base of exon per million) using RSEM version 1.1.11 (Li and Dewey, 2011). The log2‐fold changes of gene FPKM were calculated in 8‐h imbibed seeds and comparisons were made between *osipms1a* and WT. The differentially expressed genes (DEGs) with a padj (*P*‐adjusted) <0.05 were selected for further pathway analysis. Gene Ontology (GO) and KEGG pathway analyses were performed through GOseq (Young *et al*., 2010) and KOBAS (2.0) with a significant level of false discovery rate (FDR < 0.05; Xie *et al*., 2011). Three biological replications were performed.

### Samples harvested for physiological assays

Total protein, starch and sugar content were detected in the dry mature seeds. Seeds were imbibed in Petri dishes (diameter 9 cm) with 10 mL distilled water at 30 ± 1 °C for 3 days. The weight of the imbibed seeds was recorded every 12 h to calculate the moisture content of seeds. Following 0‐, 12‐, 24‐, 36‐, 48‐ and 60‐h imbibition, the seeds were harvested to detect the levels of free amino acids, GA_3_, glucose, fructose, amylase activity, acetyl‐CoA, pyruvic acid and energy. Approximately 0.5 g of each sample was rapidly frozen in liquid nitrogen and homogenized into a powder. Three biological replications were performed for each index.

### Amino acid assay

Amino acids were extracted from the powder with 2.0 mL distilled water at 4 °C for 17 h. The extraction mixture was centrifuged at 12 000 *g* at 4 °C for 20 min, following which 0.8 mL supernatant was transferred to 2‐mL Eppendorf tubes and 0.8 mL 5% 5‐sulfosalicylic acid dihydrate solution was added. After the mixture was centrifuged at 12 000 *g* at 4 °C for 20 min followed by filtration through 0.22‐μm membrane filters, the total amino acid composition was analysed using the AAA L‐8900 auto‐amino acid analyser (Hitachi Ltd., Japan). The amino acid levels were expressed as μg/g DW (dry weight).

### Hormone quantification

Hormone GA_3_ was extracted from each sample using 5 mL 80% (v/v) precooling methanol at 4 °C for 12 h and centrifuged at 8000 *g* at 4 °C for 10 min. The supernatant was collected and added 0.1 g Poly to adsorb the phenolic compounds and pigments at 4 °C for 1 h, and then, the mixture was centrifuged at 12 000 *g* at 4 °C for 10 min. Next, the supernatant was washed with 5 mL 100% (w/v) and 5 mL 80% (v/v) methanol and was passed through C18 columns (C18 Sep‐Park^®^ Cartridges, Waters Corp., Milford, MA). The extraction was freeze‐dried and dissolved in 2 mL of 75% aqueous methanol, and followed by filtration through 0.22‐μm membrane filters, the final 2 μL filtrate solution was carried out using a high‐performance liquid chromatography (HPLC) system (Waters Instruments Inc., Rochester, MN). The content of GA_3_ was determined using the external standard method and was expressed as μg/g DW.

### Protein, starch, sugar, amylase activity, acetyl‐CoA and pyruvic acid assays

Protein, starch, sugars, amylase activity, acetyl‐CoA and pyruvic acid were measured using commercial assay kits following the manufacturer's instructions (Suzhou Keming Bioengineering Company, China). The levels of protein, starch, glucose, fructose and pyruvic acid were expressed as mg/g DW. Acetyl‐CoA levels were expressed as nmol/g DW. One unit (U) of amylase is defined as 1 mg of reducing sugar produced by enzyme in 1 g DW sample in 1 min at 40 °C. The activities of α‐ and β‐amylase were expressed as U/g DW.

### Energy level assays

Extraction of adenosine triphosphate (ATP), adenosine diphosphate (ADP) and adenosine monophosphate (AMP) from the powder was conducted according to the method of Liu *et al*. (2006) with minor modifications. Briefly, 1.5 mL of 600 mm perchloric acid was added into the powder placed in an ice bath for 1 min. After the extraction mixture was centrifuged at 12 000 *g* at 4 °C for 10 min, 1.2 mL supernatant was taken and quickly neutralized to pH 6.5–6.8 with 1000 mm KOH. After the mixture was centrifuged at 12 000 *g* at 4 °C for 10 min followed by filtration through 0.22‐μm membrane filters, the final 2 μL filtrate solution was measured by HPLC (Waters Instruments Inc.). The ACQUITY UPLC^®^ BEHC18 1.7 μm 2.1 × 50 mm C18 column was used for HPLC (Waters Instruments Inc.). Identification of ATP, ADP and AMP in the samples was conducted by comparison of the retention time with that of standards. ATP, ADP and AMP levels were determined using the external standard method and were expressed as μg/g DW. The energy charge (EG) was calculated as follows: EG = ([ATP] + 1/2 [ADP]) ÷ ([ATP] + [ADP] + [AMP]).

### Seed priming

Seed priming treatments were conducted according to the method described by Cheng *et al*. (2017) with minor modifications. Briefly, 50 seeds were surface sterilized with 0.1% HgCl_2_ for 5 min and then placed in a Petri dish (diameter 9 cm) with 10 mL of distilled water. Seeds were incubated at 30 °C for 0–36 h in the dark for priming treatment, following which the imbibed seeds were dried at 30 °C for 7 days to their original moisture content (~13%). Seed germination was conducted as described above. The expression of *OsIPMS1* during seed priming was analysed by qRT‐PCR as described above. Unprimed dry seeds were used as controls. Three biological replications were performed.

### Data analysis

Experimental data were analysed using the SAS software (Cary, NC), and the percentage data were transformed according to *y* = arcsin [sqr (*x*/100)]. The significant differences were tested using Student's *t*‐test or Fisher's least significant difference (LSD) test at the 5% and 1% levels of probability.

## Availability of supporting data

The RNA sequencing data have been submitted to the Sequence Read Archive (SRA) database ( https://www.ncbi.nlm.nih.gov/sra) under accession number SRP134159.

## Conflict of interest

The authors declare no conflict of interest.

## Supporting information


**Figure S1** Characterization of *OsIPMS1* and *OsIPMS2* in rice.

**Figure S2** Comparison of amino acid sequences between OsIPMS1 and OsIPMS2.

**Figure S3** Confirmation of *osipms1b* and *osipms1c* mutants.

**Figure S4** Comparison of seed germination between WT and *osipms1* mutants under stress conditions.

**Figure S5** Comparison of seed germination between WT and *osipms1* mutants under direct‐seeding conditions.

**Figure S6** Comparison of seed germination between WT and *osipms2* mutants under normal conditions.

**Figure S7** Amino acid treatment improved the expression of GA biosynthesis related genes in germinating seeds of *osipms1* mutants.

**Figure S8** GO enrichment analysis for differentially expressed genes (DEGs) in *osipms1a* compared to WT.

**Figure S9** Comparison of imbibition rate and seed reserves between WT and *osipms1* mutants.

**Figure S10** Comparison of priming effects on seed vigor between WT and *osipms1* mutants.

**Table S1** The primer pairs used in this study.

**Table S2** Differently expression genes (DEGs) between WT and *osipms1* in 8 h‐imbibed seeds.

**Table S3** Differently expression genes (DEGs) involved in biological pathways by KEGG pathway analysis.
