# Supplementary figures and images for: Influence of isopropylmalate synthase OsIPMS1 on seed vigour associated with amino acid and energy metabolism in rice
Source: Plant Biotechnol J. 2018 Jul 16;17(2):322–37. doi: 10.1111/pbi.12979 (PMC6335077; doi:10.1111/pbi.12979)

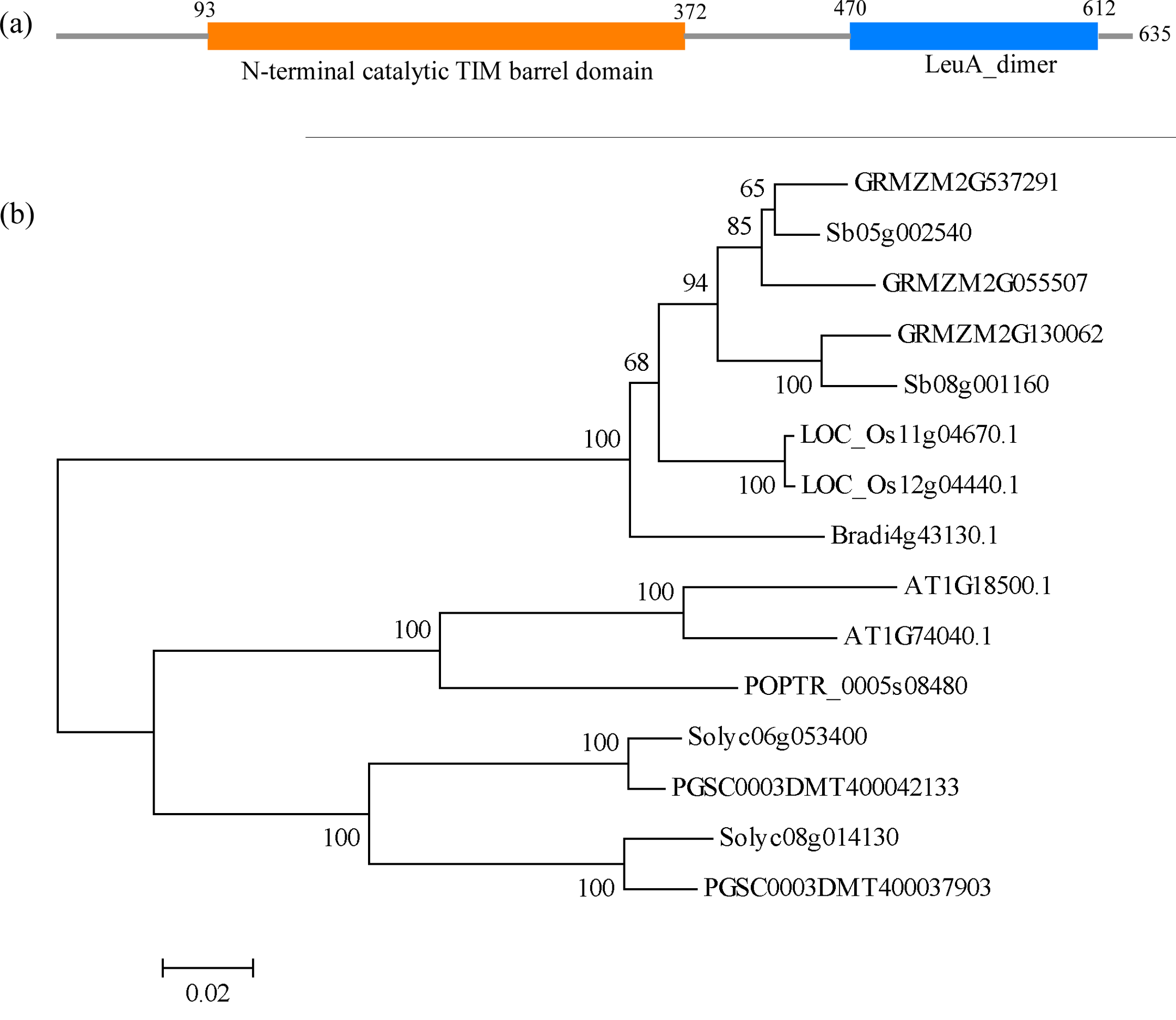

Supplement: Supplementary file 1 — Figure S1 Characterization of OsIPMS1 and OsIPMS2 in rice. [file PBI-17-322-s006.tif]

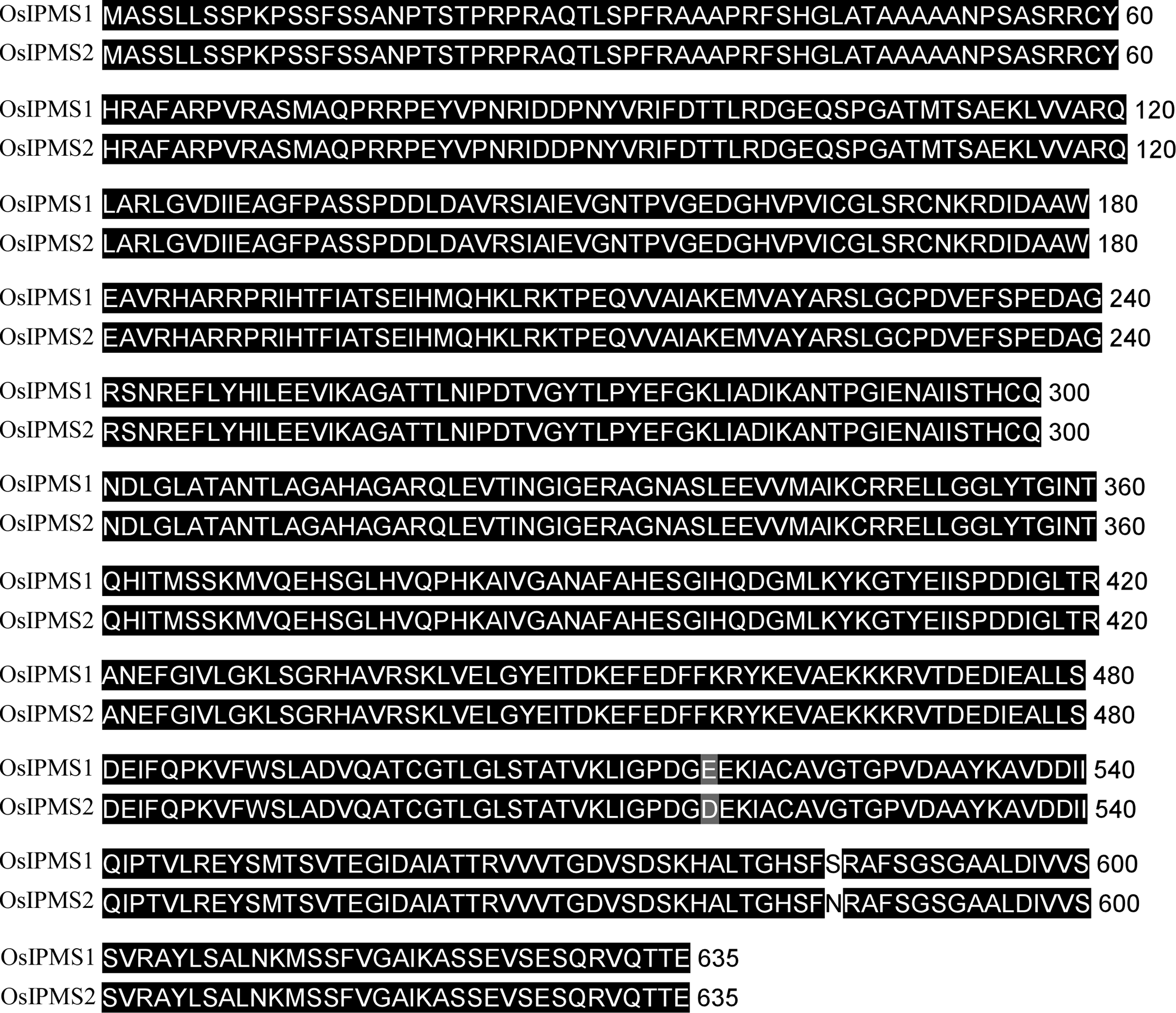

Supplement: Supplementary file 2 — Figure S2 Comparison of amino acid sequences between OsIPMS1 and OsIPMS2. [file PBI-17-322-s011.tif]

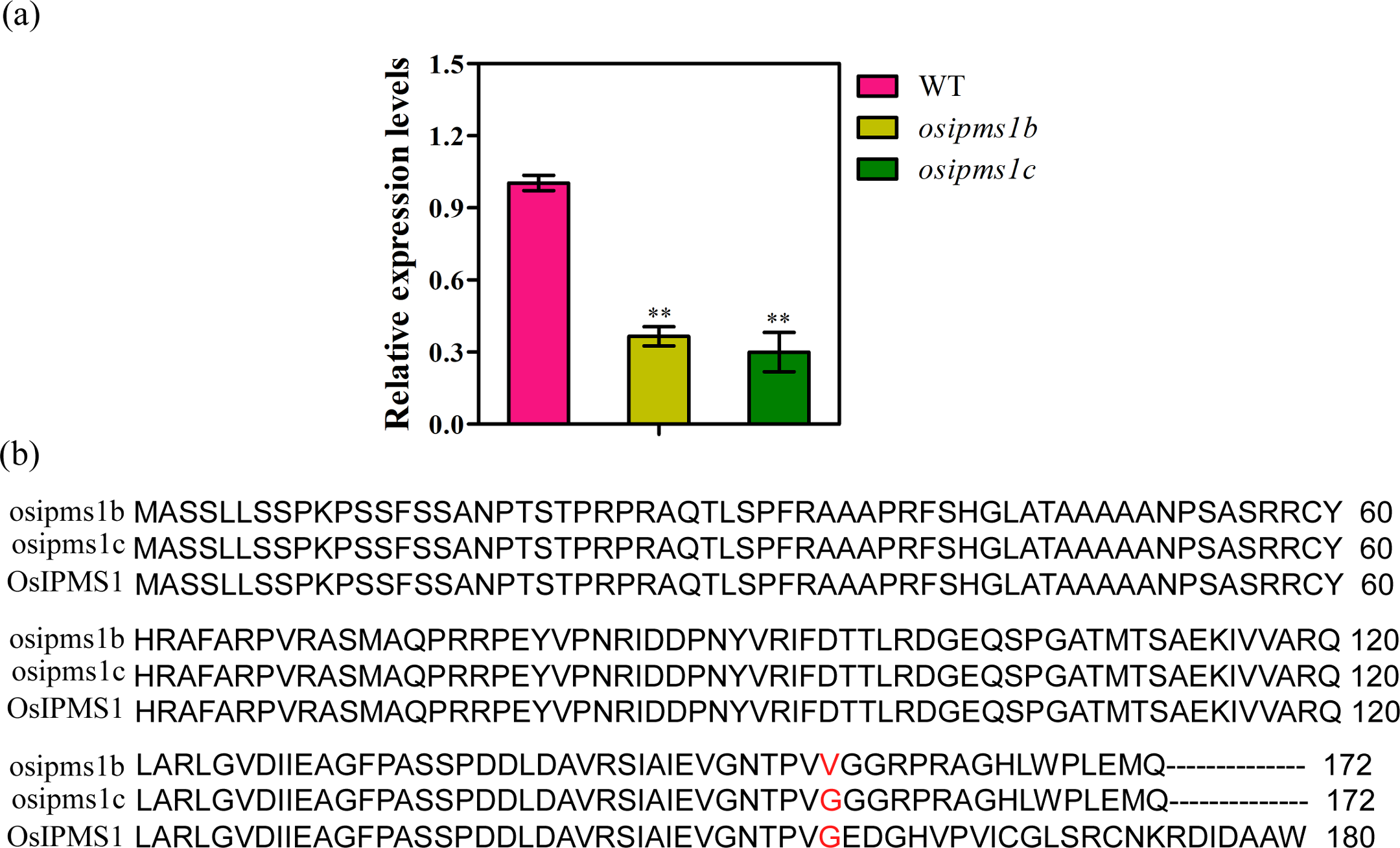

Supplement: Supplementary file 3 — Figure S3 Confirmation of osipms1b and osipms1c mutants. [file PBI-17-322-s010.tif]

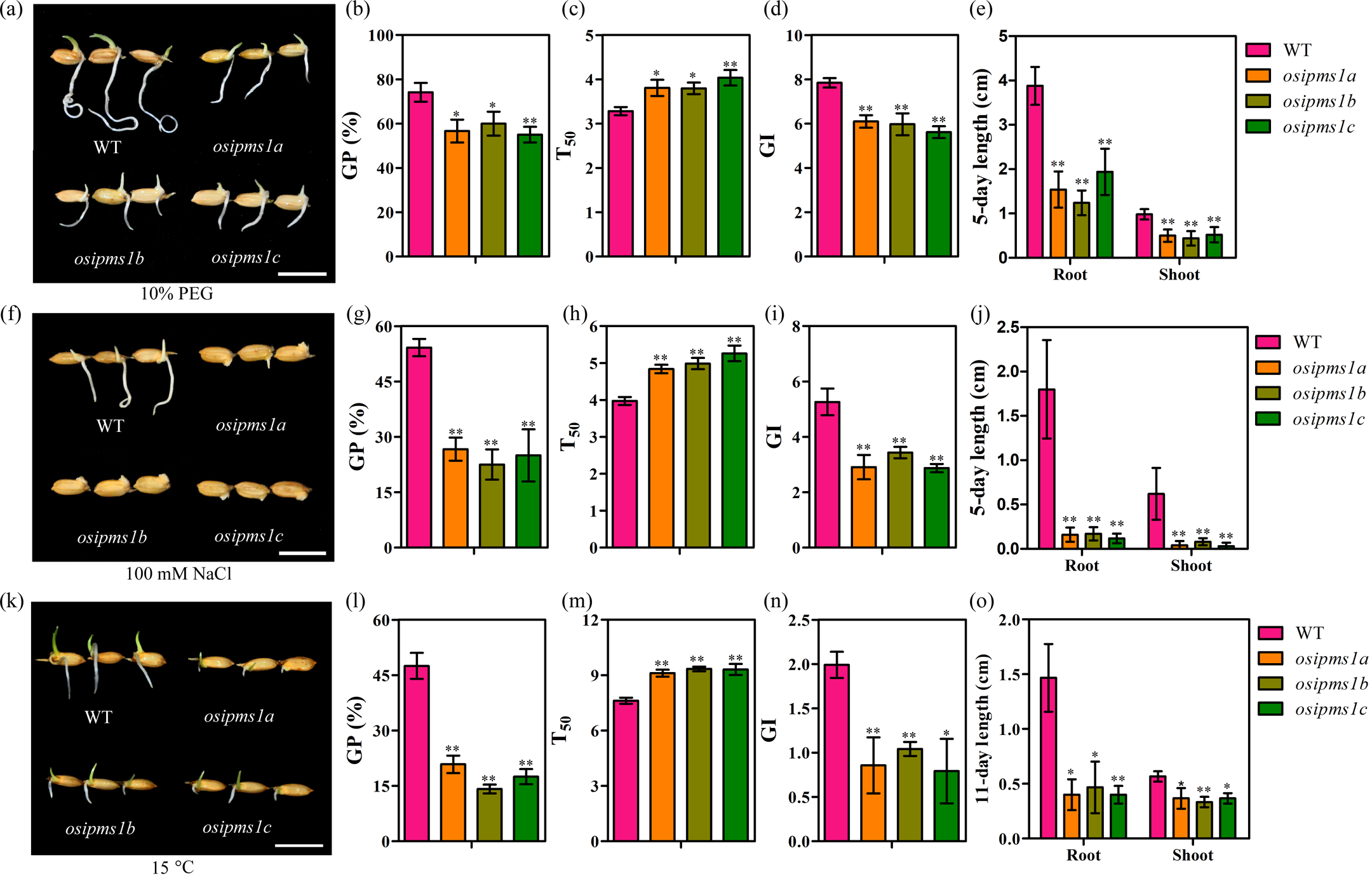

Supplement: Supplementary file 4 — Figure S4 Comparison of seed germination between WT and osipms1 mutants under stress conditions. [file PBI-17-322-s012.tif]

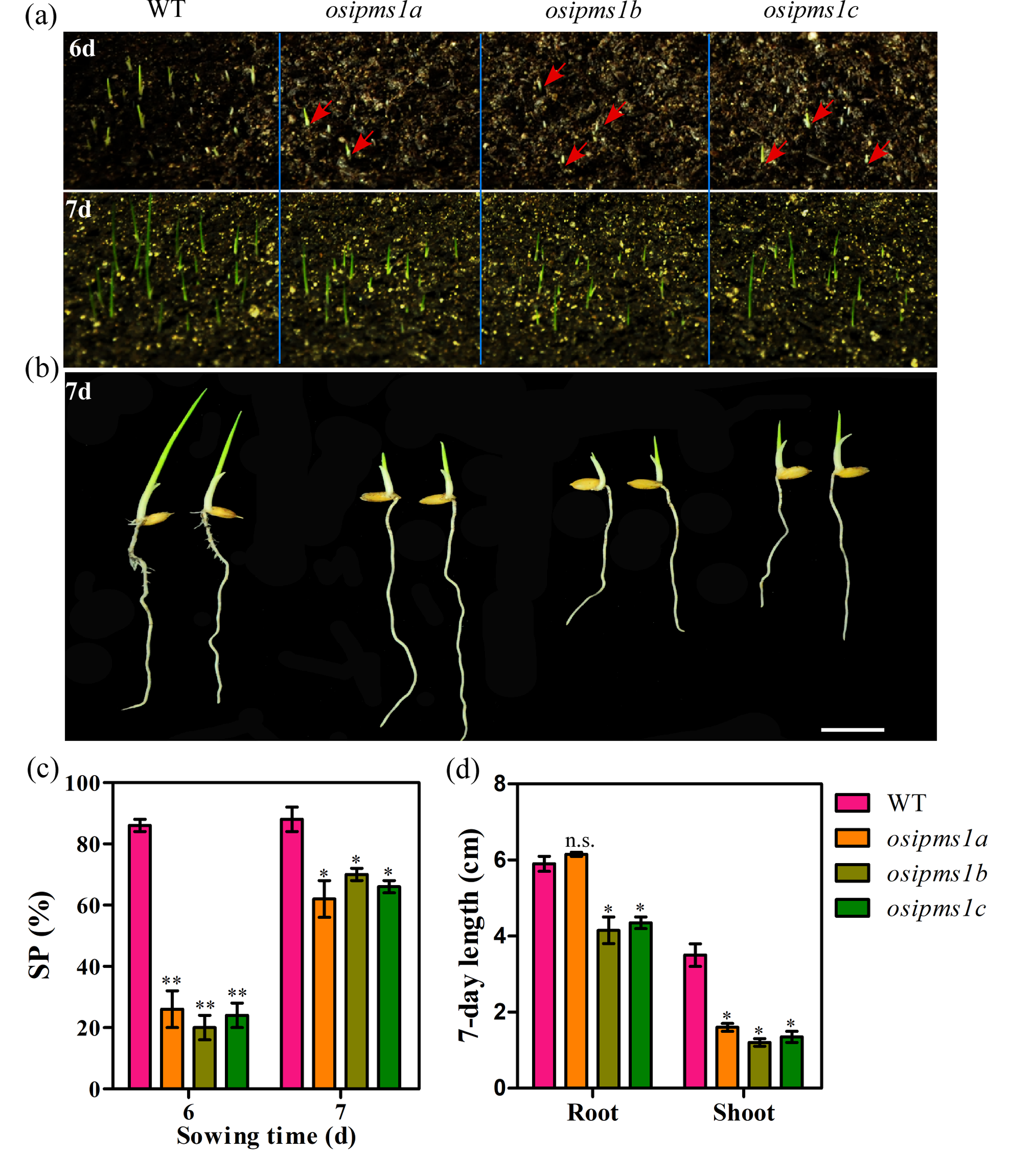

Supplement: Supplementary file 5 — Figure S5 Comparison of seed germination between WT and osipms1 mutants under direct‐seeding conditions. [file PBI-17-322-s001.tif]

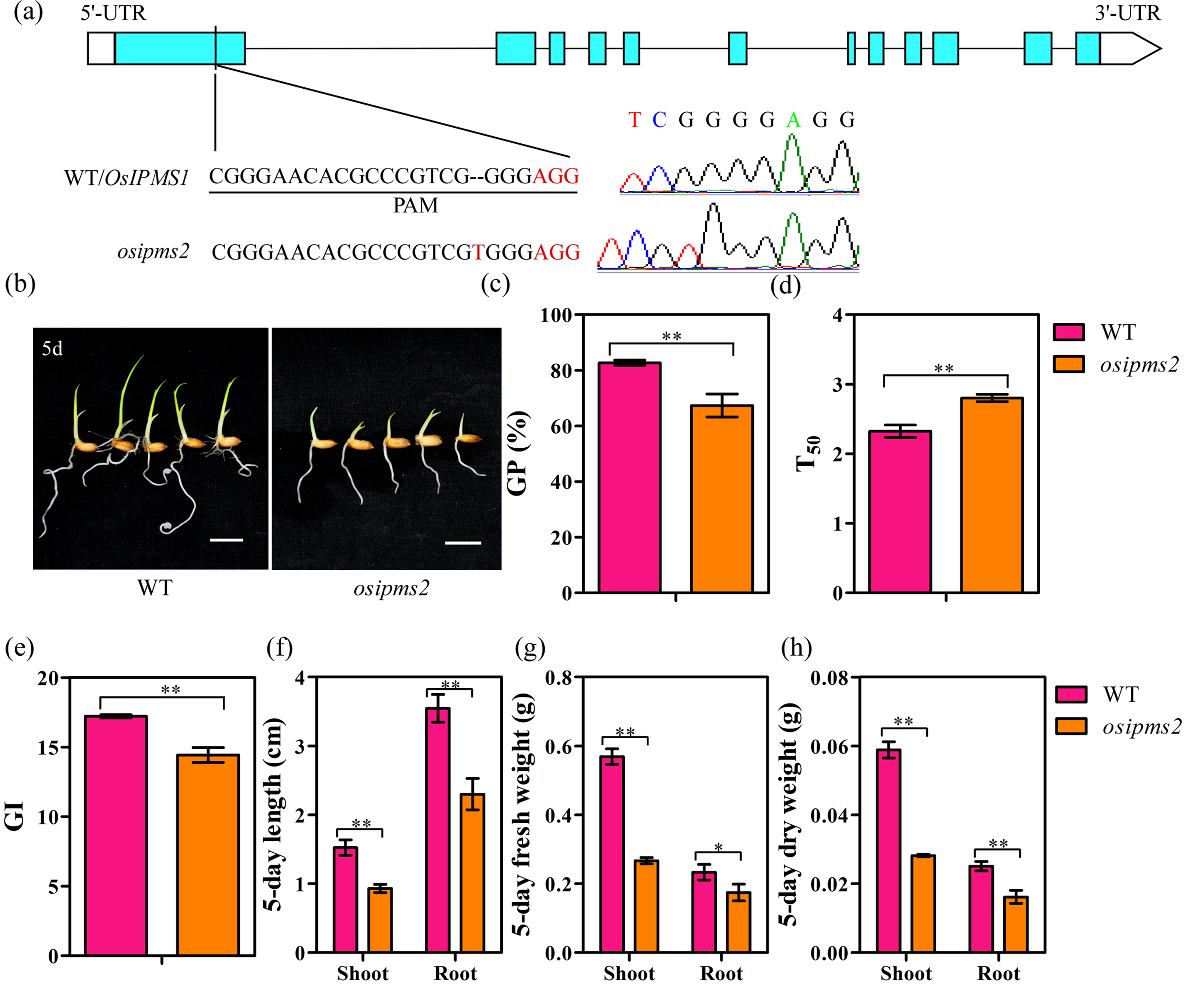

Supplement: Supplementary file 6 — Figure S6 Comparison of seed germination between WT and osipms2 mutants under normal conditions. [file PBI-17-322-s002.tif]

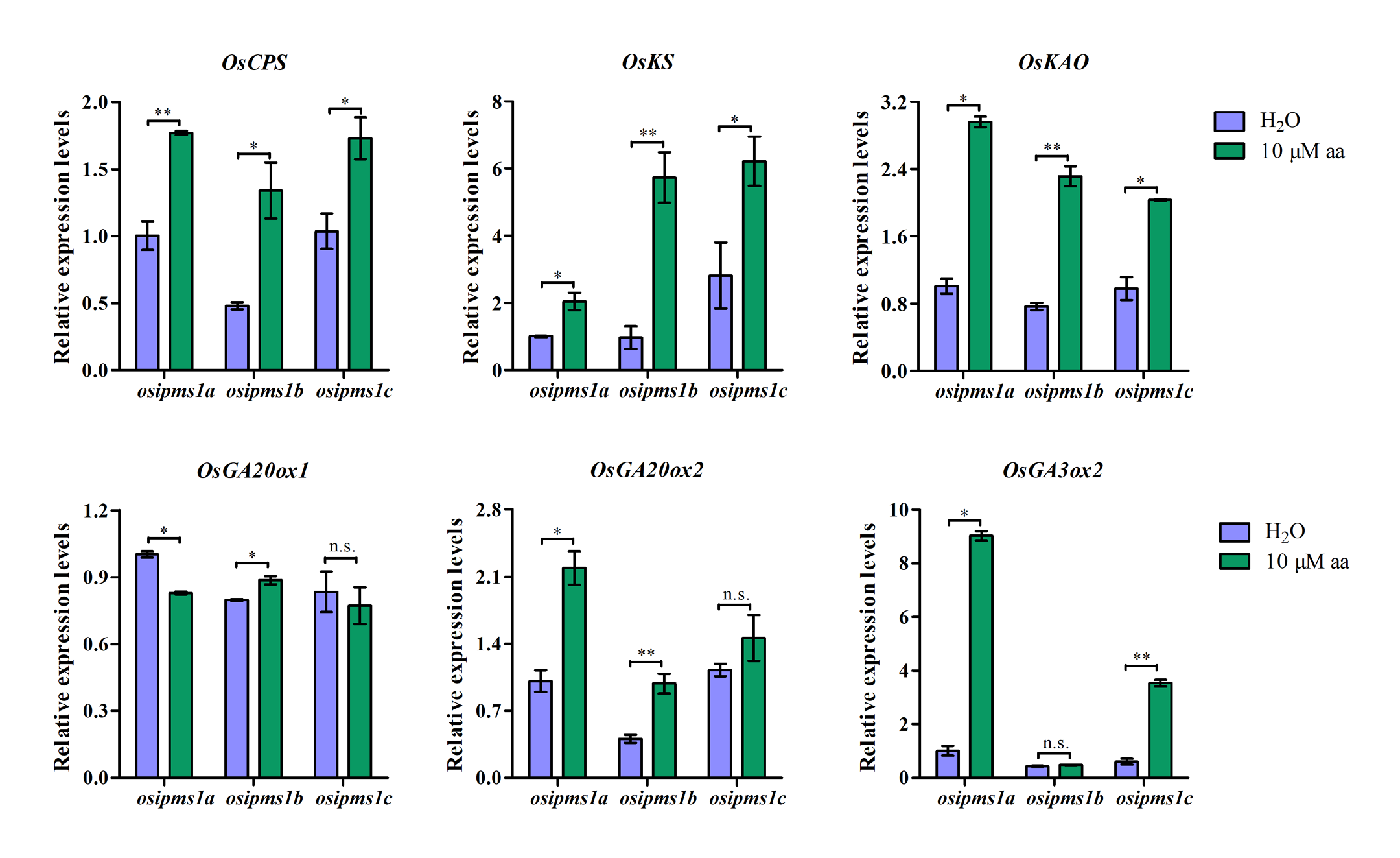

Supplement: Supplementary file 7 — Figure S7 Amino acid treatment improved the expression of GA biosynthesis related genes in germinating seeds of osipms1 mutants. [file PBI-17-322-s003.tif]

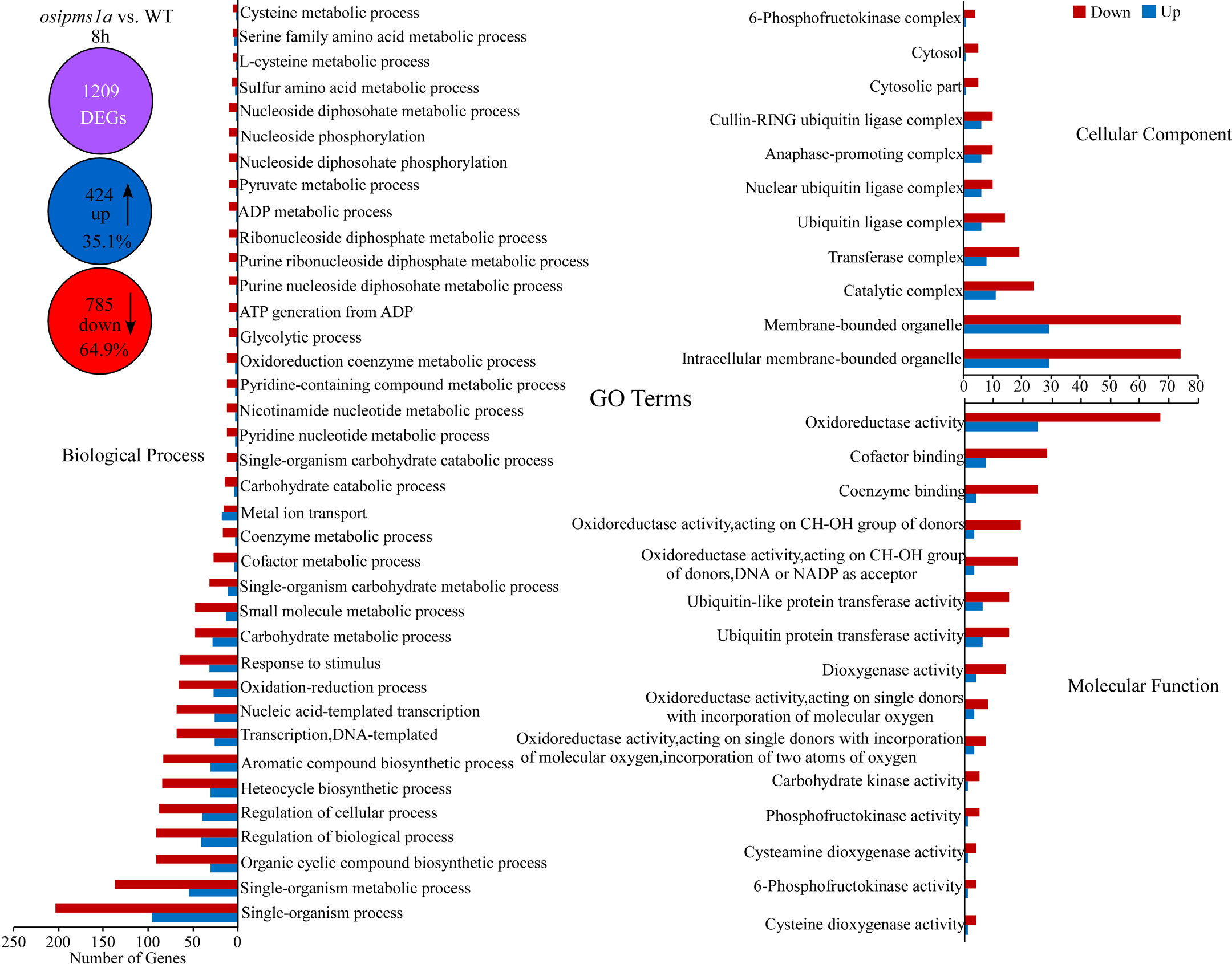

Supplement: Supplementary file 8 — Figure S8 GO enrichment analysis for differentially expressed genes (DEGs) in osipms1a compared to WT. [file PBI-17-322-s004.tif]

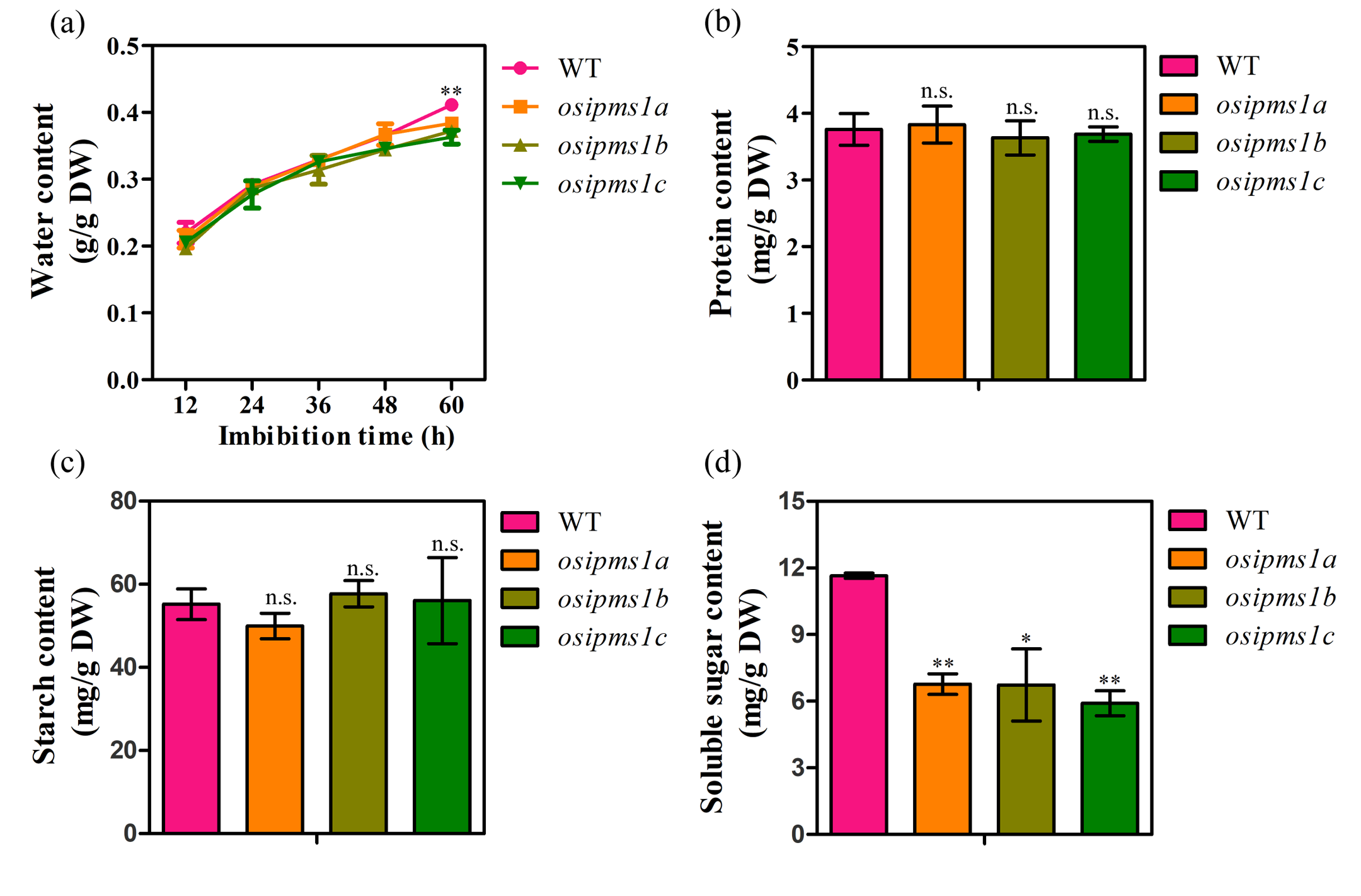

Supplement: Supplementary file 9 — Figure S9 Comparison of imbibition rate and seed reserves between WT and osipms1 mutants. [file PBI-17-322-s005.tif]

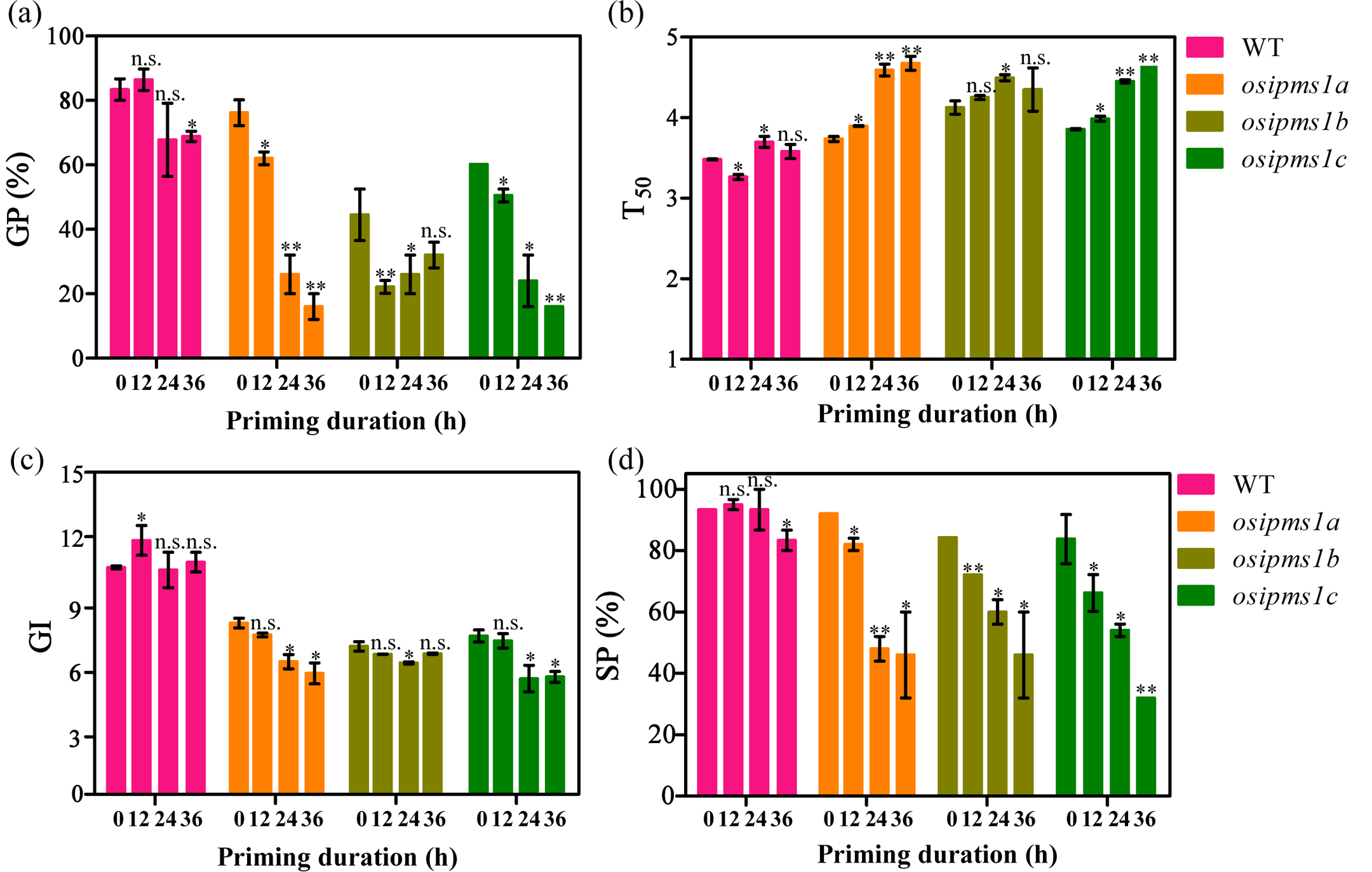

Supplement: Supplementary file 10 — Figure S10 Comparison of priming effects on seed vigor between WT and osipms1 mutants. [file PBI-17-322-s013.tif]
