# Supplementary material for: Influence of isopropylmalate synthase OsIPMS1 on seed vigour associated with amino acid and energy metabolism in rice
Source: Plant Biotechnol J. 2018 Jul 16;17(2):322–37. doi: 10.1111/pbi.12979 (PMC6335077; doi:10.1111/pbi.12979)
Supplement: Supplementary file 11 — Table S1 The primer pairs used in this study. [file PBI-17-322-s007.docx]

**Table S1.** The primer pairs used in this study

| Primer name | Sequence (5’–3’) | Purpose |
| --- | --- | --- |
| IPMS1-1S | CTACGTCCCTGCCCTTTGTACA | Cloning |
| IPMS1-1A | TCATTCTGTGGTTTGAACTCTTTGG |  |
| IPMS2-1S | CTACGTCCCTGCCCTTTGTACA | Cloning |
| IPMS2-1A | TCATTCTGTGGTTTGAACTCTTTGG |  |
| IPMS1-2S | GATCTGGTTCCGCGTGGATCCATGGCCTCCTCCCTCCTCT | GST-OsIPMS1 construction |
| IPMS1-2A | TCAGTCAGTCACGATGAATTCTCATTCTGTGGTTTGAACTCTTTGG |  |
| IPMS2-2S | GATCTGGTTCCGCGTGGATCCATGGCCTCCTCCCTCCTCT | GST-OsIPMS1 construction |
| IPMS2-2A | TCAGTCAGTCACGATGAATTCTCATTCTGTGGTTTGAACTCTTTGG |  |
| gRNA target sequences | GGGAACACGCCCGTCGGGG | gRNA target sequences |
| Tos17-L-tail | AATCCAGATCCCCCGAATTA | Mutant identification |
| LP | CACTTTCTGCTAATTTGGTG |  |
| RP | CCTTATTAAGTATGGAATCTCCTA |  |
| CRISPR1-F | ATTGCTCATGTCATGTGGTC | *osipms1* Mutant identification |
| CRISPR1-R | CTTTGAAACGCAGGATAGAA |  |
| CRISPR2-F | CACAGCATTCGTGCATCCC | *osipms2* Mutant identification |
| CRISPR2-R | GACATTTCAACGCTGACTATTT |  |
| 18S rRNA-F | CCTATCAACTTTCGATGGTAGG | qRT-PCR |
| 18S rRNA-R | CGTTAAGGGATTTAGATTGTAC |  |
| OsActin-F | AGGAAGGCTGGAAGAGGACC | qRT-PCR |
| OsActin-R | CGGGAAATTGTGAGGGACAT |  |
| OsIPMS1-F | GCCGAGAGCTCTTAGGAGGTCT | qRT-PCR |
| OsIPMS1-R | TTTCCCAAGAACAATACCAAACTCG |  |
| OsIPMS2-F | CGTCGAGAACTCTTAGGAGGTCTGTA | qRT-PCR |
| OsIPMS2-R | CTTCCCAAGAACAATACCAAACTCA |  |
| OsPFK1-F | GCCGGCTATAGCTTCACTGT | qRT-PCR |
| OsPFK1-R | TGTTGGCCGTCCTATCGTTC |  |
| OsPFK2-F | GCTGCTGCTCCCAAGAAGAA | qRT-PCR |
| OsPFK2-R | GTACTGCTTGACGACGGAGT |  |
| OsPFK3-F | CTGTTGTCCTCGACCAACCA | qRT-PCR |
| OsPFK3-R | CACCATTTGCCGGCTTCTTT |  |
| OsPK1-F | TGCAAGGGGTGATTTGGGAA | qRT-PCR |
| OsPK1-R | CTAGTAGGGCGGGGAGACTT |  |
| OsPK2-F | GGGCTTGCTAAGGCTCTGAA | qRT-PCR |
| OsPK2-R | AGGGTGAACAGTGAACCAGC |  |
| OsPK3-F | TTGCTAAGGCTGTGAAGCCA | qRT-PCR |
| OsPK3-R | AATGTGAAGAGCGACCCAGC |  |
| OsPK4-F | GACATGGGGAAGATCCTGGC | qRT-PCR |
| OsPK4-R | GAGGGTCTCCTGGTGGTACT |  |
| OsPEPCK-F | TCTAACATTGAGGGCGGCTG | qRT-PCR |
| OsPEPCK-R | GCAGCACGGGTATTCTCTGT |  |
| OsCPS-F | GCGTGCATTTTCGAACCAA | qRT-PCR |
| OsCPS-R | TTGGCCAGCACTGACACTCT |  |
| OsKS-F | AGCTGTTCTGGAAGATGTGC | qRT-PCR |
| OsKS-R | CTTGACGACTGCATTCACTG |  |
| OsKO-F | CGGTGACTACCAAAAGATGG | qRT-PCR |
| OsKO-R | ATGTGGGTCAAGAGAAACCA |  |
| OsKAO-F | TTAGCTGGAACCGAAAACAG | qRT-PCR |
| OsKAO-R | ACCAAAGCGAGCATATCAAG |  |
| OsGA20ox1-F | GCCACTACAGGGCCGACAT | qRT-PCR |
| OsGA20ox1-R | TGGTTGCAGGTGACGATGAT |  |
| OsGA20ox2-F | CCAATTTTGGACCCTACCGC | qRT-PCR |
| OsGA20ox2-R | GAGAGAAGCCCAACCCAACC |  |
| OsGA3ox2-F | TCCTCCTTCTTCTCCAAGCTC | qRT-PCR |
| OsGA3ox2-R | GAAACTCCTCCATCACGTCAC |  |
