# Supplementary material for: Influence of isopropylmalate synthase OsIPMS1 on seed vigour associated with amino acid and energy metabolism in rice
Source: Plant Biotechnol J. 2018 Jul 16;17(2):322–37. doi: 10.1111/pbi.12979 (PMC6335077; doi:10.1111/pbi.12979)
Supplement: Supplementary file 13 — Table S3 Differently expression genes (DEGs) involved in biological pathways by KEGG pathway analysis. [file PBI-17-322-s009.docx]

**Table S3.** Differently expression genes (DEGs) involved in biological pathways by KEGG pathway analysis

| Term | Sample number | Background number | Corrected P-value | UniGenes |
| --- | --- | --- | --- | --- |
| Glycolysis / Gluconeogenesis | 27 | 119 | 3.50E-05 | LOC_Os09g15820 LOC_Os03g15950 LOC_Os01g46950 LOC_Os05g39310 LOC_Os05g10650 LOC_Os10g13700 LOC_Os05g44922 LOC_Os10g08550 LOC_Os03g21260 LOC_Os04g33190 LOC_Os01g16960 LOC_Os12g05110 LOC_Os02g38920 LOC_Os01g67860 LOC_Os01g53680 LOC_Os05g39320 LOC_Os01g60190 LOC_Os08g03290 LOC_Os04g58110 LOC_Os08g34210 LOC_Os06g40640 LOC_Os11g10510 LOC_Os03g18220 LOC_Os05g33380 LOC_Os01g62420 LOC_Os11g05110 LOC_Os02g49720 |
| Protein processing in endoplasmic reticulum | 31 | 203 | 0.0032 | LOC_Os03g57340 LOC_Os11g09280 LOC_Os03g16030 LOC_Os05g38530 LOC_Os07g43180 LOC_Os03g14180 LOC_Os08g39140 LOC_Os01g62290 LOC_Os04g36750 LOC_Os03g16920 LOC_Os07g43240 LOC_Os06g11610 LOC_Os01g46926 LOC_Os05g23140 LOC_Os07g43260 LOC_Os06g14240 LOC_Os01g62244 LOC_Os03g60780 LOC_Os04g01740 LOC_Os03g16020 LOC_Os02g52150 LOC_Os03g16040 LOC_Os02g54140 LOC_Os01g04360 LOC_Os12g14070 LOC_Os03g16860 LOC_Os07g43250 LOC_Os01g04340 LOC_Os05g23740 LOC_Os02g48140 LOC_Os11g13980 |
| Pyruvate metabolism | 13 | 75 | 0.0304 | LOC_Os12g05110 LOC_Os11g05110 LOC_Os08g33720 LOC_Os11g04670 LOC_Os04g58110 LOC_Os10g13700 LOC_Os02g49720 LOC_Os05g09440 LOC_Os03g31750 LOC_Os04g33190 LOC_Os05g49880 LOC_Os01g16960 |
| Carbon metabolism | 29 | 225 | 0.0374 | LOC_Os06g05700 LOC_Os03g15950 LOC_Os05g49880 LOC_Os05g10650 LOC_Os02g38840 LOC_Os10g13700 LOC_Os05g44922 LOC_Os03g60090 LOC_Os10g08550 LOC_Os05g09440 LOC_Os04g33190 LOC_Os01g16960 LOC_Os12g05110 LOC_Os02g38920 LOC_Os03g21260 LOC_Os01g67860 LOC_Os04g58110 LOC_Os08g33720 LOC_Os01g60190 LOC_Os01g55540 LOC_Os03g31750 LOC_Os08g03290 LOC_Os01g53680 LOC_Os08g34210 LOC_Os06g40640 LOC_Os10g25130 LOC_Os05g33380 LOC_Os01g62420 LOC_Os11g05110 |
| Fructose and mannose metabolism | 11 | 53 | 0.0374 | LOC_Os06g40640 LOC_Os05g10650 LOC_Os01g67860 LOC_Os06g13810 LOC_Os05g33380 LOC_Os01g53680 LOC_Os05g44922 LOC_Os01g62420 LOC_Os08g25720 LOC_Os06g22060 LOC_Os03g16150 |
| Taurine and hypotaurine metabolism | 6 | 16 | 0.0374 | LOC_Os03g45210 LOC_Os09g29480 LOC_Os12g42860 LOC_Os03g13300 LOC_Os03g45250 LOC_Os08g37470 |
